# Effect modification and interaction between ethnicity and socioeconomic factors in severe COVID-19: analyses of linked national data for Scotland

**DOI:** 10.1136/bmjopen-2024-092727

**Published:** 2025-04-14

**Authors:** Ronan McCabe, Eliud Kibuchi, Sarah Amele, Patricia Irizar, Aziz Sheikh, Karen Jeffrey, Igor Ruden, Colin R Simpson, Colin McCowan, Lewis Ritchie, Chris Robertson, Alastair H Leyland, Evangelia Demou, Anna Pearce, Srinivasa Vittal Katikireddi

**Affiliations:** 1 MRC/CSO Social and Public Health Sciences Unit, University of Glasgow, Glasgow, UK; 2 Department of Sociology, The University of Manchester, Manchester, UK; 3 Nuffield Department of Primary Care Health Sciences, University of Oxford, Oxford, UK; 4 Usher Institute, The University of Edinburgh, Edinburgh, UK; 5 Victoria University of Wellington, Wellington, Wellington, New Zealand; 6 University of St Andrews, St Andrews, UK; 7 General Practice and Primary Care, University of Aberdeen, Aberdeen, UK; 8 Department of Mathematics and Statistics, University of Strathclyde, Glasgow, Glasgow, UK

**Keywords:** COVID-19, EPIDEMIOLOGIC STUDIES, EPIDEMIOLOGY

## Abstract

**Abstract:**

**Objective:**

Minority ethnic groups disproportionately experienced adverse COVID-19 outcomes, partly a consequence of disproportionate exposure to socioeconomic disadvantage and high-risk occupations. We examined whether minority ethnic groups were also disproportionately vulnerable to the consequences of socioeconomic disadvantage and high-risk occupations in Scotland.

**Design:**

We investigated effect modification and interaction between area deprivation, education and occupational risk and ethnicity (assessed as both a binary white vs non-white variable and a multi-category variable) in relation to severe COVID-19 (hospitalisation or death). We used electronic health records linked to the 2011 census and Cox proportional hazards models, adjusting for age, sex and health board. We were principally concerned with additive interactions as a measure of vulnerability, estimated as the relative excess risk due to interaction (RERI).

**Results:**

Analyses considered 3 730 837 individuals aged ≥16 years (with narrower age ranges for analyses focused on education and occupation). Severe COVID-19 risk was typically higher for minority ethnic groups and disadvantaged socioeconomic groups, but additive interactions were not consistent. For example, non-white ethnicity and highest deprivation level experienced elevated risk ((HR=2.7, 95% CI: 2.4, 3.2) compared with the white least deprived group. Additive interaction was not present (RERI=−0.1, 95% CI: −0.4, 0.2), this risk being less than the sum of risks of white ethnicity/highest deprivation level (HR=2.4, 95% CI: 2.3, 2.5) and non-white ethnicity/lowest deprivation level (1.4, 95% CI: 1.2, 1.7). Similarly, non-white ethnicity/no degree education (HR=2.5, 95% CI: 2.2, 2.7; RERI=−0.1, 95% CI: −0.4, 0.2) and non-white ethnicity/high-risk occupation (RERI=0.3, 95% CI: −0.2, 0.8) did not experience greater than additive risk. No clear evidence of effect modification was identified when using the multicategory ethnicity variable or on the multiplicative scale either.

**Conclusion:**

We found no definitive evidence that minority ethnic groups were more vulnerable to the effect of social disadvantage on the risk of severe COVID-19.

**STRENGTHS AND LIMITATIONS OF THIS STUDY**

- Data on socioeconomic characteristics linked to individual health records.
- Almost complete population coverage.
- Records potentially outdated leading to misclassification.
- Low case numbers for certain ethnic subgroups.

## Background

 Ethnicity is an important dimension of health inequalities, with differences in morbidity and mortality having been repeatedly observed across ethnic groups for a range of acute and chronic conditions.^1^,^3^ This has demonstrably been the case during the COVID-19 pandemic where minoritised and racialised ethnic groups have disproportionately experienced adverse outcomes across different geographical contexts.^4^,^6^ These ethnic inequalities are understood to be driven by racist social processes operating at different levels (eg, interpersonal, institutional and structural) which, in the most immediate sense, influence exposure to SARS-CoV-2 infection and subsequent vulnerability to developing severe forms of COVID-19.^7^,^9^

Other dimensions of social position have also been associated with differential impacts during the COVID-19 pandemic, where the most socioeconomically disadvantaged groups and certain occupations have experienced the highest rates of adverse outcomes.^10^,^12^ Previous empirical research has indicated that differential exposure to socioeconomic disadvantage among minority ethnic groups accounts, in part, for ethnic inequalities in health more broadly and there are indications that this is similarly the case for ethnic inequalities in adverse COVID-19 outcomes specifically.^1 13 14^ Similarly, individuals from minoritised ethnic groups were more likely to be employed in occupations with a high risk of exposure to SARS-CoV-2 infection (eg, health and social care).^14^

Ethnic inequalities in adverse COVID-19 might also arise through differential vulnerability to socioeconomic disadvantage or high-risk occupations. That is, the health consequences of these exposures may be greater in some ethnic groups than others due to, for example, the presence of comorbidities, allostatic load or reduced ability to self-isolate.^7 8^ However, while studies have considered differential exposure to socioeconomic disadvantage and high-risk occupations in understanding ethnic differences in health, differential vulnerability to these factors has so far received limited attention.^13 14^

In this article, we investigate whether some ethnic groups were more vulnerable to the impacts of socioeconomic disadvantage and occupation on severe COVID-19 (hospitalisation or death) than others in Scotland.

## Methods

### Population

We considered all individuals who were alive, ≥16 years old, present in both the 2011 Scottish Census and Community Health Index (CHI) register, and resident in Scotland on 1 March 2020 (date of the first laboratory confirmed SARS-CoV-2 infection in Scotland), which was determined based on the CHI register. For analyses using education as an effect modifier, this population was age-restricted to those aged ≥30 years (on the above date) so that all individuals would have potentially completed tertiary education at the time of recording in 2011 and for analyses using occupation as an effect modifier, this population was age-restricted to those aged between 30 and 64 years as these individuals were of working age both in 2020 and in 2011 when occupation data were recorded. A flow diagram detailing the formation of these different study populations is presented in online supplemental material figure S1. Those recorded as economically inactive in the census were excluded, although economic inactivity was more prevalent among minority ethnic groups (online supplemental table S1).

### Data

We used data from the Early Pandemic Evaluation and Enhanced Surveillance of COVID-19 (EAVE-II) platform linked to data from the 2011 Scottish Census. The EAVE-II platform includes virological, vaccination, primary care, hospitalisation and mortality data for around 99% of the Scottish population.^15 16^ We used the following data sets from EAVE-II: COVID-19 test data for SARS-CoV-2 testing data, Scottish Morbidity Record 01 for hospitalisations, National Records of Scotland death registry (deaths) and CHI register for data on area deprivation, age, sex and health board. The census provided data on occupation and education, which were not present in Scottish health records. Ethnicity data were also derived from the census; although ethnicity data were present in Scottish health records, the census is considered the gold standard, with a recent study indicating considerable misclassification of ethnicity in Scottish health records.^17^

### Outcome

Our outcome of interest was COVID-19 hospitalisation or death (hereafter referred to as severe COVID-19). In line with previous studies, COVID-19 hospitalisation was defined based on the International Classification of Diseases (ICD) 10 code (U07.1 and U07.2) listed in any diagnostic position or a hospitalisation where the individual had a positive reverse transcription PCR (RT-PCR) test for SARS-CoV-2 in the 28 days prior to admission.^17 18^ A COVID-19 death was defined as having ICD-10 code (U07.1, U07.2) recorded as the primary or secondary causes of death on the death certificate, or death from any cause within 28 days of a positive SARS-CoV-2 RT-PCR test.

### Exposure

Ethnicity, our exposure variable, was aggregated both to a binary variable (white vs non-white) and a multicategory variable with six levels (white Scottish; white other British or Irish; other white; South Asian; African, Caribbean or black; and other ethnicity). For the multicategory variable, we modified the standard 2011 census five-level aggregation in light of low case numbers of our outcome across levels of each effect modifier (see online supplemental table S2 for details).

### Effect modifiers

We considered three measures of socioeconomic disadvantage—area deprivation and education occupational risk as effect modifiers of the association between ethnicity and severe COVID-19. We define effect modification here simply as observed differences in the outcome across combinations of the exposure and the effect modifier.^19^ Area deprivation was measured using the 2020 Scottish Index of Multiple Deprivation (SIMD) data obtained from the CHI register.^16^ SIMD ranks small ‘data zones’ in Scotland (composed of~700 individuals) by level of deprivation measured across seven weighted domains (employment, health, education, housing, income, service access and crime across the region). We used SIMD quintiles, with quintile 1 indicating the most deprived 20% of data zones and quintile 5 the least deprived 20% data zones. While health is one of the domains within SIMD, it makes a small contribution to the overall index and previous work has shown that this results in minimal bias. Education level was classified as a binary variable (degree vs no degree). We classified occupation into three categories based on likelihood of SARS-CoV-2 exposure (low-risk, medium-risk and high-risk). These categories were adapted from previously published classifications^20^ by five authors (EK, RM, AP, ED and SVK) who independently assessed infection and mortality risks using three-digit standardised occupational classification (2010) codes. Where there was a discrepancy, authors discussed and reached a consensus on the appropriate level of risk (see online supplemental table S3).

### Model confounders

**Figure 1:**
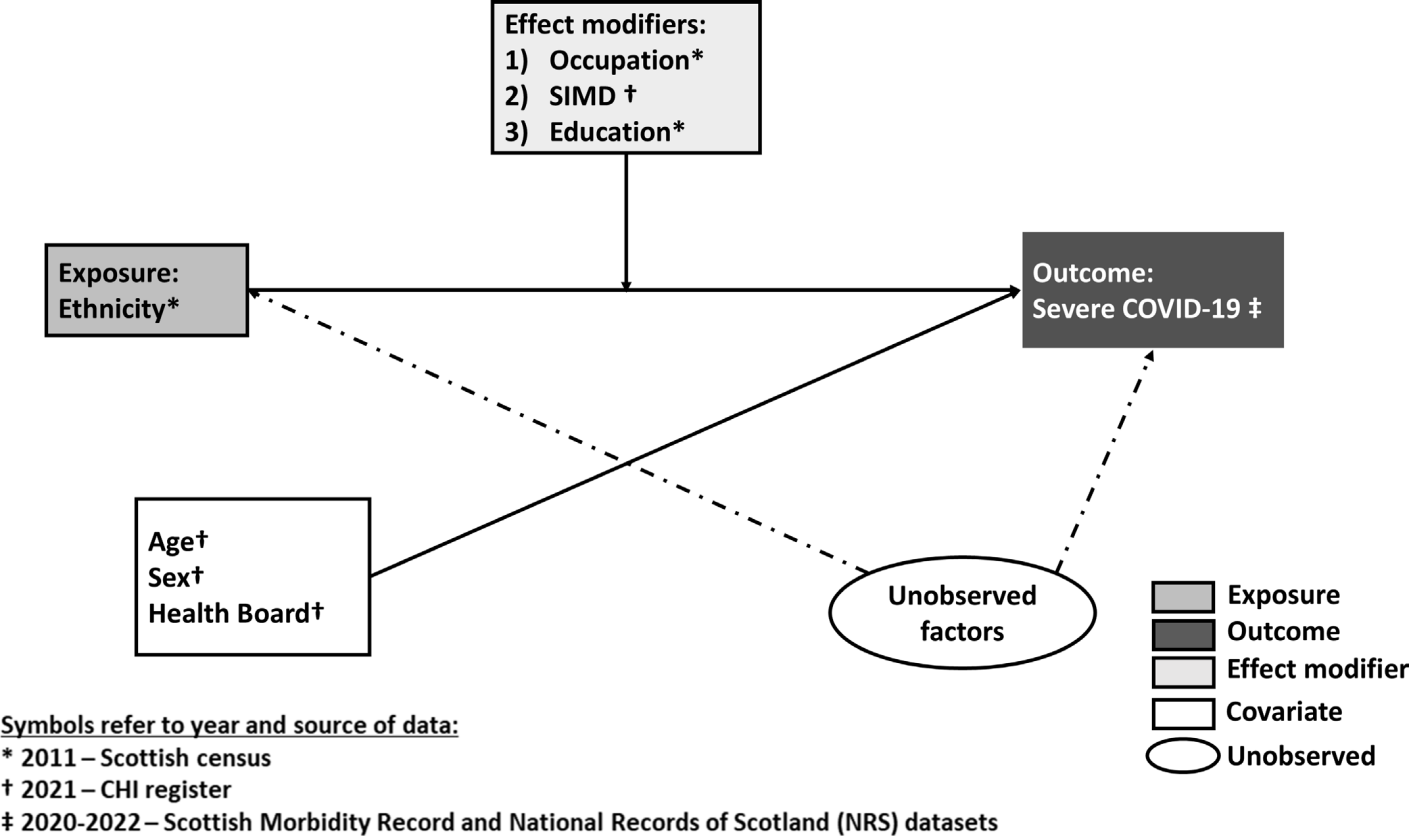
Directed acyclic graph representing the relationship between COVID-19, ethnicity, effect modifiers (occupation, SIMD, education) and confounders (age, sex and health board). Each effect modifier was explored in a separate model. CHI, Community Health Index; SIMD, Scottish Index of Multiple Deprivation.

Age (as a restricted cubic spline with 4 df) as of March 2020, sex and health board (regional authorities responsible for health service delivery) were prespecified confounders for all analyses. The directed acyclic graph (figure 1) presents the relationship between outcome, exposure, effect modifiers and confounders.

### Statistical analyses

We used Cox proportional hazard models to estimate the risk of severe COVID-19 since the time for the first COVID-19 hospitalisation or death was the outcome of interest. Using calendar time, we followed individuals from 1 March 2020 (date of first SARS-CoV-2 case in Scotland) until the first of: experiencing the outcome, death from any cause, or 30 April 2022. Models provided HRs with a 95% CI. Models used a shared reference category which, for area deprivation, education level and occupational risk were, respectively, the white group (or white Scottish group when using the multicategory measure of ethnicity) in the least deprived SIMD quintile (SIMD=5), with a degree and in low-risk occupations. This allowed us to see how the risk of severe COVID-19 varied across combinations of the effect modifiers and ethnicity.^21^ We considered interactions on the additive (relative excess risk due to interaction or RERI) and multiplicative (ratio of HRs) scales. The RERI estimates the absolute difference in HRs between different groups, whereas multiplicative interactions refer to the relative difference in HRs between different groups. We were principally interested in the former as it is considered to be most relevant to public health.^19^ A positive interaction at a higher level compared with a lower level disadvantage would correspond to higher vulnerability and a negative interaction to lower vulnerability. We used a Monte Carlo approach to estimate CIs for all interactions. This involved sampling 1000 times from the distribution of the relative parameters (means and variances/covariances) to derive the interaction measure (eg, RERI), and taking the 2.5 and 97.5 percentiles of the distribution for this measure as its lower and upper intervals, respectively.

We conducted secondary analyses for the analyses examining interactions between ethnicity and occupation, stratifying by pandemic wave, as occupational practices in response to the pandemic changed over our observation period. These were defined following previous studies as wave 1 (1 March 2020 to 31 July 2020), wave 2 (1 August 2020 to 30 April 2021), wave 3 (1 May 2021 to 17 December 2021) and wave 4 (18 December 2021 to 30 April 2022), which were informed by changes in reproduction, growth and positivity rates.^18^

### Patient

Patient and Public Involvement (PPI) for this research extended from the broader PPI undertaken in conjunction with the EAVE-II platform.

## Results

There were 5 121 530 individuals in the EAVE-11 data set, 51 138 of which did not have data on health board and 1 339 555 who did not have disaggregated ethnicity data and were excluded from the analysis. We included 3 730 837 individuals ≤16 years old in analyses using area deprivation as effect modifier (online supplemental tables S4 and S5), 3 060 250 individuals ≥30 years old in analyses using education level as effect modifier (online supplemental table S6), and 2 000 556 individuals between 30 and 64 years old in analyses using occupational risk (online supplemental table S7). Overall, these populations were broadly similar in composition across ethnicity by SIMD, sex and health board. However, they differed slightly by education level and occupation due to differences in the percentage of missing values (online supplemental table S8). We now detail findings from analyses of each effect modifier.

### Ethnicity and area deprivation

**Figure 2:**
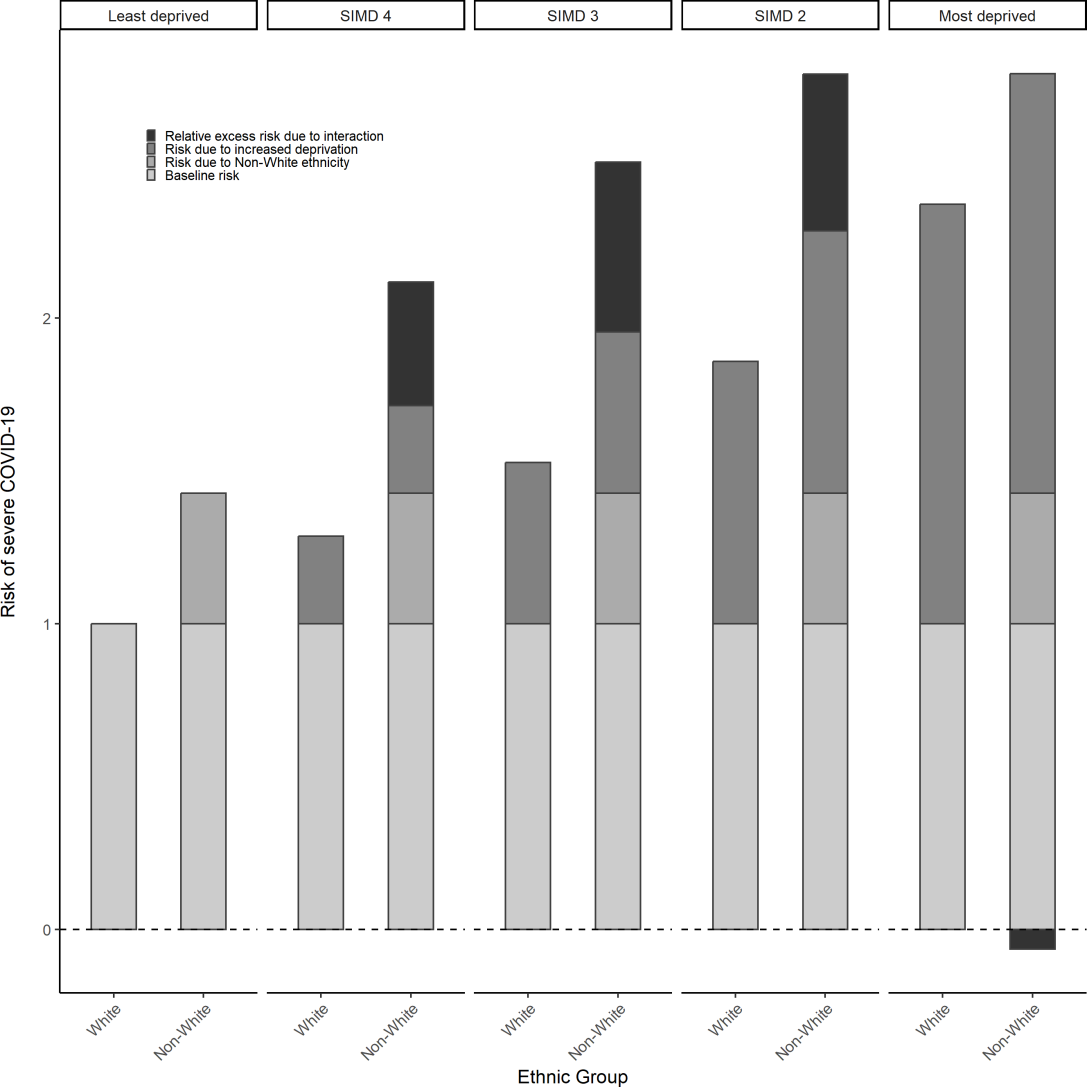
Risk of severe COVID-19 (hospitalisation or death) by ethnicity across levels of area deprivation (Scottish Index of Multiple Deprivation or SIMD quintiles, where SIMD 5=least deprived and SIMD 1=most deprived).

The proportion of individuals across levels of deprivation was similar for the white group (19.2–20.4%), however showed greater range for the non-white group, with the highest proportion in the least deprived SIMD quintile (16.3–25.4%) (online supplemental material, table S4). Table 1 shows the HRs for severe COVID-19 outcomes across combinations of ethnicity and deprivation. White individuals living in the most deprived areas were 2.4 times more likely to experience severe COVID-19 outcomes compared with white individuals in the least deprived areas (HR=2.373, 95% CI: 2.293, 2.456). Non-white individuals also carried an elevated risk compared with white individuals. For example, compared with least deprived white individuals, least deprived non-white individuals were 1.4 times more likely to experience severe COVID-19 outcomes (HR=1.427, 95% CI: 1.215, 1.676). Evidence of differential vulnerability, however, was mixed. For example, the risk among non-white individuals who were also living in the most deprived areas was slightly less than the sum of the two combined risk factors previously described (with an RERI of −0.065, 95% CI: −0.497, 0.377). In contrast, positive additive interactions (ie, the combined risks were greater than the sum of the parts) were seen for the middle deprivation groups (with RERIs ranging from 0.4 to 0.56), although also with wide CIs. Similar patterns were observed on the multiplicative scale, although effect sizes were small (table 1). Figure 2 visualises the risk in non-white individuals and more socioeconomically deprived individuals, when experienced separately and combined, as compared with white, advantaged individuals with estimates presented in online supplemental table S9.

**Table 1:** Risk of severe COVID-19 (hospitalisation or death) across effect modifiers (Scottish Index of Multiple Deprivation or SIMD, education level and occupational risk) within binary ethnic groupings

Area deprivation					
Ethnicity	SIMD 5(least deprived)	SIMD 4	SIMD 3	SIMD 2	SIMD 1(most deprived)
	HR (95% CI)	HR (95% CI)	HR (95% CI)	HR (95% CI)	HR (95% CI)
White	1	1.287(1.23, 1.336)	1.528(1.473, 1.586)	1.859(1.796, 1.925)	2.373(2.293, 2.456)
Non-white	1.427(1.215, 1.676)	2.118(1.82, 2.464)	2.511(2.139, 2.946)	2.798(2.418, 3.237)	2.735(2.375, 3.15)
Additive interaction (RERI)		0.404(−0.002, 0.799)	0.556(0.119, 1.015)	0.512(0.044, 0.977)	−0.065(−0.497, 0.377)
Multiplicative interaction (ratio of HRs)		1.153(0.921, 1.43)	1.152(0.916, 1.432)	1.055(0.843, 1.296)	0.808(0.653, 0.984)
Education					
Ethnicity	Degree	No degree			
	HR (95% CI)	HR (95% CI)			
White	1	1.812(1.76, 1.866)			
Non-white	1.745(1.54, 1.978)	2.451(2.246, 2.676)			
Additive interaction (RERI)		−0.106(−0.401, 0.173)			
Multiplicative interaction (ratio of HRs)		0.775(0.669, 0.898)			
Occupation					
Ethnicity	Low risk	Medium risk	High risk		
	HR (95% CI)	HR (95% CI)	HR (95% CI)		
White	1	1.556(1.488, 1.626)	1.11(1.047, 1.178)		
Non-white	1.534(1.254, 1.876)	2.073(1.807, 2.377)	1.938(1.573, 2.387)		
Additive interaction (RERI)		−0.017(−0.435, 0.368)	0.293(−0.175, 0.800)		
Multiplicative interaction (ratio of HRs)		0.869(0.686, 1.099)	1.138(0.863, 1.529)		

RERI, relative excess risk due to interaction.

Results are based on three separate models on three different study populations exploring each effect modifier separately.

When looking at the more detailed ethnic classification, we also found that higher levels of deprivation corresponded to elevated risk of severe COVID-19, particularly for South Asian and African, Caribbean or black individuals compared with white Scottish individuals (see table 2). However, there was no clear pattern in terms of differential vulnerability to the combined effects of deprivation and minority ethnic status on severe COVID-19 (see table 2). For example, negative additive interactions were present across all deprivation levels for white British and Irish individuals (compared with white Scottish in the least deprived areas). Conversely, positive additive interactions were present for African, Caribbean or black and South Asian individuals across all levels of deprivation, excepting those exposed to the highest level of deprivation for the latter (RERI=−0.048, 95% CI: −0.728, 0.661). Interaction on the multiplicative scale was broadly equivalent in direction, although diverged, for example, among white other British or Irish individuals exposed to higher levels of deprivation (see table 2). Figure 2 visualises the risk of severe COVID-19 experienced in non-white individuals, individuals with lower education and the excess risk for experiencing both, as compared with white individuals with a degree (see online supplemental table S9).

**Table 2:** Risk of severe COVID-19 (hospitalisation or death) across effect modifiers (Scottish Index of Multiple Deprivation or SIMD, education level and occupational risk) within ethnic groupings. Results are based on three separate models exploring each effect modifier separately

Area deprivation					
Ethnicity	SIMD 5	SIMD 4	SIMD 3	SIMD 2	SIMD 1
	HR (95% CI)	HR (95% CI)	HR (95% CI)	HR (95% CI)	HR (95% CI)
White Scottish	1	1.281(1.231, 1.333)	1.522(1.465, 1.582)	1.819(1.754, 1.887)	2.325(2.243, 2.41)
White British or Irish	0.73(0.662, 0.804)	0.93(0.847, 1.02)	1.03(0.939, 1.129)	1.466(1.332, 1.613)	1.974(1.79, 2.177)
Other white	0.779(0.61, 0.996)	1.026(0.802, 1.314)	1.356(1.088, 1.689)	1.637(1.344, 1.995)	1.623(1.346, 1.957)
South Asian	1.79(1.477, 2.168)	2.647(2.213, 3.168)	2.983(2.439, 3.648)	3.658(3.067, 4.362)	3.067(2.497, 3.767)
African, Caribbean or black	1.013(0.506, 2.027)	1.532(0.823, 2.849)	2.257(1.359, 3.747)	2.075(1.306, 3.297)	2.598(1.923, 3.509)
Other ethnicity	0.885(0.646, 1.213)	1.279(0.94, 1.739)	1.733(1.283, 2.34)	1.62(1.195, 2.195)	2.24(1.757, 2.857)
Additive interaction (RERI)					
White British or Irish		−0.081(−0.188, 0.041)	−0.222(−0.345 to –0.11)	−0.083(−0.234, 0.071)	−0.081(−0.268, 0.145)
Other white		−0.034(−0.347, 0.304)	0.055(−0.302, 0.419)	0.039(−0.32, 0.422)	−0.481(−0.826 to –0.135)
South Asian		0.576(−0.019, 1.216)	0.671(0.043, 1.371)	1.049(0.349, 1.753)	−0.048(−0.728, 0.661)
African, Caribbean or black		0.238(−0.878, 1.713)	0.722(−0.521, 2.172)	0.243(−0.979, 1.571)	0.26(−0.873, 1.224)
Other ethnicity		0.113(−0.393, 0.598)	0.326(−0.216, 0.968)	−0.084(−0.626, 0.505)	0.03(−0.541, 0.649)
Multiplicative interaction (ratio of HRs)					
White British or Irish		0.995(0.874, 1.143)	0.927(0.811, 1.051)	1.104(0.97, 1.255)	1.163(1.026, 1.34)
Other white		1.028(0.726, 1.452)	1.144(0.819, 1.587)	1.155(0.865, 1.583)	0.896(0.666, 1.191)
South Asian		1.154(0.882, 1.519)	1.095(0.839, 1.431)	1.123(0.853, 1.43)	0.737(0.558, 0.959)
African, Caribbean or black		1.181(0.493, 2.974)	1.464(0.658, 3.446)	1.126(0.491, 2.612)	1.103(0.538, 2.265)
Other ethnicity		1.128(0.712, 1.732)	1.287(0.836, 2)	1.006(0.647, 1.521)	1.089(0.731, 1.62)
Education					
Ethnicity	Degree	No degree			
	HR (95% CI)	HR (95% CI)			
White Scottish	1	1.773(1.718, 1.829)			
White British or Irish	0.782(0.722, 0.847)	1.349(1.274, 1.428)			
Other white	0.86(0.725, 1.021)	1.671(1.479, 1.886)			
South Asian	1.965(1.656, 2.333)	2.967(2.671, 3.295)			
African, Caribbean or black	1.82(1.348, 2.458)	2.056(1.517, 2.787)			
Other ethnicity	1.31(1.05, 1.634)	1.546(1.301, 1.835)			
Additive interaction (RERI)					
White British or Irish		−0.206(−0.301, –0.11)			
Other white		0.038(−0.211, 0.283)			
South Asian		0.229(−0.232, 0.663)			
African, Caribbean or black		−0.537(−1.388, 0.268)			
Other ethnicity		−0.537(0.937, –0.125)			
Multiplicative interaction (ratio of HRs)					
White British or Irish		0.973(0.89, 1.07)			
Other white		1.096(0.892, 1.344)			
South Asian		0.852(0.701, 1.048)			
African, Caribbean or black		0.637(0.411, 0.959)			
Other ethnicity		0.666(0.504, 0.904)			
Occupation					
Ethnicity	Low risk	Medium risk	High risk		
	HR (95% CI)	HR (95% CI)	HR (95% CI)		
White Scottish	1	1.548(1.478, 1.621)	1.128(1.061, 1.199)		
White British or Irish	0.725(0.638, 0.824)	1.054(0.915, 1.215)	0.694(0.576, 0.836)		
Other white	0.756(0.585, 0.976)	1.409(1.181, 1.68)	0.825(0.568, 1.197)		
South Asian	1.682(1.264, 2.238)	2.756(2.342, 3.243)	1.526(1.058, 2.2)		
African, Caribbean or black	1.548(0.961, 2.494)	2.02(1.328, 3.073)	2.037(1.339, 3.099)		
Other ethnicity	1.251(0.888, 1.763)	1.029(0.762, 1.39)	2.15(1.574, 2.937)		
Additive interaction (RERI)					
White British or Irish		−0.219(−0.388, –0.044)	−0.159(−0.322, 0.012)		
Other white		0.105(−0.2, 0.429)	−0.059(−0.406, 0.372)		
South Asian		0.526(−0.179, 1.137)	−0.284(−0.99, 0.568)		
African, Caribbean or black		−0.076(−1.228, 1.201)	0.361(−0.823, 1.542)		
Other ethnicity		−0.77(−1.319 to –0.224)	0.771(0.053, 1.582)		
Multiplicative interaction (ratio of HRs)					
White British or Irish		0.939(0.786, 1.119)	0.849(0.679, 1.06)		
Other white		1.204(0.902, 1.65)	0.967(0.608, 1.565)		
South Asian		1.058(0.76, 1.472)	0.804(0.512, 1.307)		
African, Caribbean or black		0.843(0.457, 1.54)	1.167(0.628, 2.214)		
Other ethnicity		0.531(0.346, 0.852)	1.524(0.991, 2.356)		

RERI, relative excess risk due to interaction.

### Ethnicity and education level

**Figure 3:**
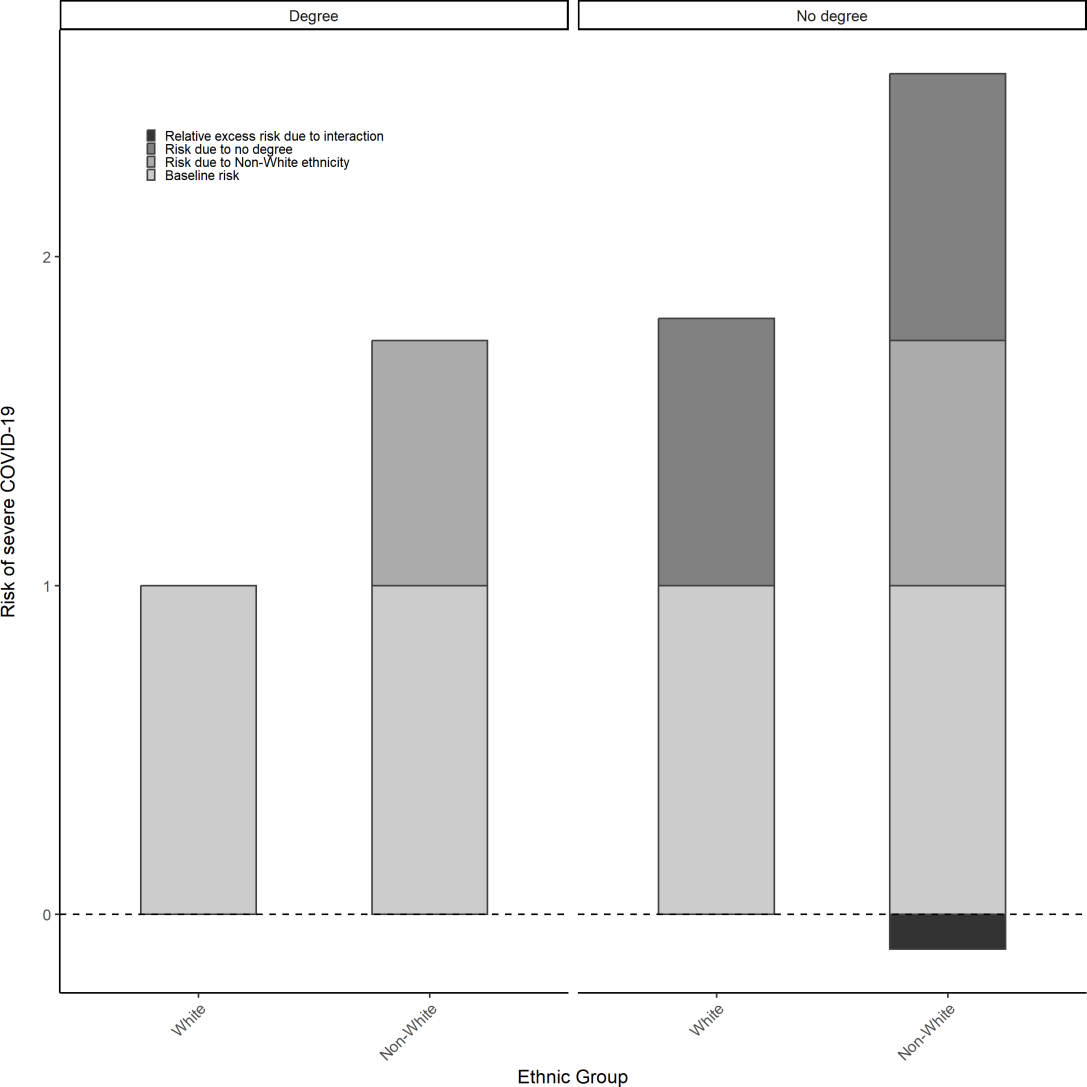
Risk of severe COVID-19 (hospitalisation or death) by ethnicity across levels of education.

For both white and non-white groups, most individuals were without degree level education (72.2% and 51.9%, respectively) (online supplemental table S6). Compared with white individuals with a degree, the risk of severe COVID-19 was 1.7 times higher for non-white individuals with a degree (HR=1.745, 95% CI: 1.540, 1.978) and 1.8 times higher among white individuals without a degree (HR=1.812, 95% CI: 1.76, 1.866) (see online supplemental table S10). However, despite non-white individuals with a degree experiencing an elevated risk (HR=2.45, 95% CI: 2.246, 2.676), there was a negative additive interaction, although CIs were wide (RERI=−0.106, 95% CI: −0.401, 0.173) (see table 2 and figure 3).

Using the multicategory ethnicity measure, the proportion of individuals without degree level education was highest among the white Scottish group (75.0%) and lowest among the African, Caribbean or black group (44.4%) (online supplemental table S5). For all ethnic groups, the risk of severe COVID-19 was higher among individuals without a degree (table 2). Compared with white Scottish individuals with a degree, risk was highest among South Asian (HR=2.967, 95% CI: 2.671, 3.295) and African Caribbean, or black (HR=2.056, 95% CI: 1.517, 2.787) individuals and lowest among white other British or Irish individuals (HR=1.349, 95% CI: 1.274, 1.428), without a degree (table 2). However, evidence of differential vulnerability was again unclear, with negative additive interactions present for white other British (RERI=−0.206, 95% CI: −0.301, –0.11) and African, Caribbean or black (RERI=−0.537, 95% CI: −1.388, 0.268) individuals without a degree and positive additive interaction present for South Asian individuals without a degree (RERI=0.229, 95% CI: −0.232, 0.663); and 95% CIs were wide for these estimates.

Results are based on three separate models exploring each effect modifier separately.

### Ethnicity and occupational risk

**Figure 4:**
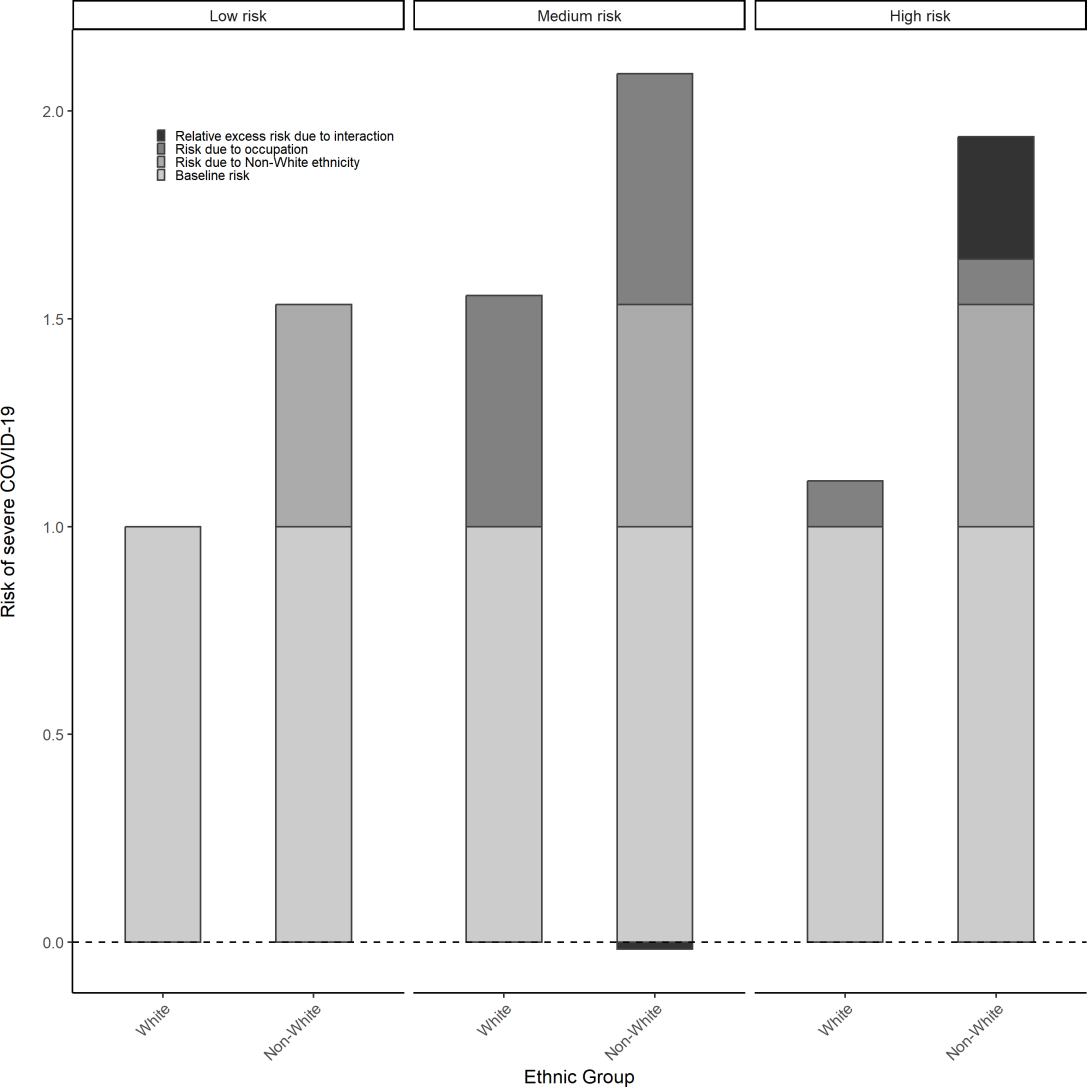
Risk of severe COVID-19 (hospitalisation or death) by ethnicity across levels of occupational risk.

There was a notably higher proportion of non-white individuals in medium-risk occupations compared with white individuals (45.8% vs 30.0%) (online supplemental table S7). Compared with white individuals in the lower occupation groups, the risk of severe COVID-19 was highest for both white (HR=1.556, 95% CI: 1.488, 1.626) and non-white (HR=2.073, 95% CI: 1.807, 2.377) individuals in medium-risk occupations and higher for non-white individuals across all levels of occupational risk (table 1). There was a positive additive interaction between white/non-white ethnicity and high/low occupational risk, although estimate CIs were wide (RERI=0.293, 95% CI: −0.175, 0.800) (displayed graphically in figure 4). Using the multicategory ethnicity measure, the African, Caribbean or black group had the highest proportion of individuals working in high-risk occupations (30.5%) and the white other British or Irish group had the highest in low-risk occupations (48.6%) (see online supplemental table S11).

As with the binary measure of ethnicity, compared with the white Scottish individuals in low-risk occupations, the risk of severe COVID-19 was elevated among those in medium-risk occupations; notably, this was the case for white other British or Irish and other white groups, despite being at a reduced risk in high-risk occupations (table 2). There was no evidence of differential vulnerability on the additive scale. For example, South Asian individuals experienced an excess risk of severe COVID-19 in medium-risk occupations (RERI=0.526, 95% CI: −0.179, 1.137), whereas African, Caribbean or black individuals experienced an excess risk in high-risk occupations (RERI=0.361, 95% CI: −0.823, 1.542), although CIs were again wide (see table 2 and online supplemental table S12).

Stratified analyses by pandemic wave indicated that the risk of severe COVID-19 was highest in high-risk occupations only for the initial two waves of the pandemic (online supplemental tables S12 and tS13), (online supplemental tables S14 and S15).

## Discussion

We found that ethnic inequalities in the risk of severe COVID-19 persisted across strata of area deprivation, educational level and occupational risk, with minority ethnic groups typically experiencing an elevated risk compared with the majority population. Increased social disadvantage—higher levels of deprivation and no degree education—and medium-risk/high-risk occupations corresponded to elevated risk in all ethnic groupings. However, there was no clear evidence that minority ethnic groups were more vulnerable to the consequences of disadvantageous socioeconomic conditions. For example, while positive additive interactions were observed for non-white individuals experiencing mid-level deprivation, those exposed to the highest level of deprivation did not appear to be more vulnerable than white individuals despite experiencing a higher risk (HR=2.735, 95% CI: 2.375, 3.150; RERI=−0.065, 95% CI: −0.497, 0.377). Educational level similarly indicated that non-white individuals were not more vulnerable to the effects of not having a degree, despite experiencing higher overall risk (HR=2.45, 95% CI: 2.246, 2.676; RERI=−0.106, 95% CI: −0.401, 0.173). There was some indication that non-white individuals—and specifically African, Caribbean or black individuals—were more vulnerable to the consequences of being in high-risk occupations, although these estimates lacked precision. Multiplicative measures of interaction often corresponded to their additive counterparts in terms of effect direction.

We add to previous research suggesting that socioeconomic disadvantage and occupation operate alongside ethnicity in shaping the risk-averse COVID-19 outcomes, and draw specific attention to the possibility that vulnerability to these factors (rather than, or in addition to, exposure) may differ by ethnicity.^13 22^ Our findings also highlight the necessity of having high-quality and population-level ethnicity data alongside both socio-demographic data and health data.^17^ For measures like occupation, despite being of particular importance with respect to COVID-19, research exploring combined effects with ethnicity has been limited.^12 14 23^ On occupation, counter to what might be expected, we found that the association between occupational risk and severe COVID-19 was often highest among those in medium-risk occupations. Although, given that we also found risk to be highest in high-risk occupations at the beginning of the pandemic, this may reflect the adoption of protective measures in high-risk occupations across the pandemic or that those who survived towards the later stages of the pandemic were less vulnerable to developing severe COVID-19.

Our study has benefitted from having data on socioeconomic characteristics linked to individual health records. Importantly, this offers almost complete population coverage, avoiding collider bias that can arise in more restricted population samples.^24^ However, there are some methodological limitations. It is worth noting that data on ethnicity, education and occupation were captured in the 2011 census and may have changed by the time of study. As such, this may have resulted in biased estimates. However, data for area deprivation were based on the CHI register (which is continually updated) rather than the 2011 census and therefore have less potential for bias arising from misclassification. Additionally, low case numbers prevented the use of more disaggregated ethnic groupings, potentially concealing important heterogeneity.^17^ The wide CIs observed across estimates may suggest that low case numbers remained a source of imprecision with the multicategory ethnicity measure used in our analyses. It can also be noted that the interpretation of findings is shaped by our choice of reference group for ethnicity, with differing conclusions possible if a group other than white Scottish (eg, white British or Irish) were chosen instead. Moreover, missingness of more than 5% in the occupational risk among minority ethnic groups may have resulted in biased estimates.

In conclusion, while socioeconomic disadvantage and occupation influenced the risk of severe COVID-19, ethnic inequalities persisted across levels of these effect modifiers. However, we did not find consistent evidence that minority ethnic groups were more vulnerable to the effect of social disadvantage or occupation on the risk of severe COVID-19.

## Supplementary material

10.1136/bmjopen-2024-092727 online supplemental file 1

## Data Availability

Data may be obtained from a third party and are not publicly available.
