# Supplementary material for: Effect modification and interaction between ethnicity and socioeconomic factors in severe COVID-19: analyses of linked national data for Scotland
Source: BMJ Open. 2025 Apr 14;15(4):e092727. doi: 10.1136/bmjopen-2024-092727 (PMC11997826; doi:10.1136/bmjopen-2024-092727)
Supplement: online supplemental file 1 [file bmjopen-15-4-s001.docx]

**Supplemental Material: Effect modification and interaction between ethnicity and socio-economic factors in severe COVID-19: analyses of linked national data for Scotland**

**
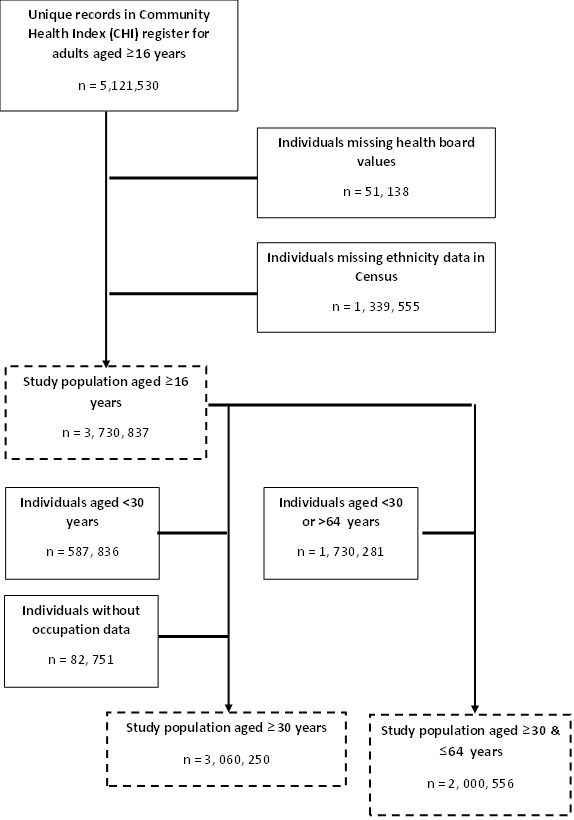
**

**Figure S1:** The flow diagram detailing the formation of these different study populations

**Table S1**: Population characteristics for the three study populations used in analysis. Age at the start of the follow-up period (March 1^st^, 2020).

|  | Individuals  (aged ≥16 years) | | Individuals  (aged ≥30 years) | | Individuals  (aged ≥30 & ≤64) | |
| --- | --- | --- | --- | --- | --- | --- |
|  | n | % | n | % | n | % |
| **Ethnic group** | | | | | | |
| White | 3624737 | 97.2 | 2980239 | 97.4 | 1940535 | 97.0 |
| Non-white | 106100 | 2.8 | 80011 | 2.6 | 60021 | 3.0 |
| *TOTAL* | 3730837 | 100.0 | 3060250 | 100.0 | 2000556 | 100.0 |
| White Scottish | 3224104 | 86.4 | 2624352 | 85.8 | 1698226 | 84.9 |
| White British or Irish | 309736 | 8.3 | 277226 | 9.1 | 175326 | 8.8 |
| Other White | 90897 | 2.4 | 78661 | 2.6 | 66983 | 3.3 |
| South Asian | 48950 | 1.3 | 36696 | 1.2 | 26836 | 1.3 |
| African, Caribbean or Black | 14729 | 0.4 | 11502 | 0.4 | 9477 | 0.5 |
| Other | 42421 | 1.1 | 31813 | 1.0 | 23708 | 1.2 |
| **Education level** | | | | | | |
| No qualification | 778435 | 20.9 | 757162 | 24.7 | 265751 | 13.3 |
| GCSE or equivalent | 813292 | 21.8 | 700326 | 22.9 | 528530 | 26.4 |
| A-level or equivalent | 833676 | 22.3 | 735084 | 24.0 | 579228 | 29.0 |
| Degree | 871802 | 23.4 | 867678 | 28.4 | 627047 | 31.3 |
| Missing | 433632 | 11.6 | NA | | 0 | 0.0 |
| **Scottish Index of Multiple Deprivation** | | | | | | |
| 1 (most deprived) | 718406 | 19.3 | 574758 | 18.8 | 381414 | 19.1 |
| 2 | 748964 | 20.1 | 611792 | 20.0 | 400613 | 20.0 |
| 3 | 757143 | 20.3 | 626504 | 20.5 | 403941 | 20.2 |
| 4 | 761895 | 20.4 | 631337 | 20.6 | 415341 | 20.8 |
| 5 (least deprived) | 744429 | 20.0 | 615859 | 20.1 | 399247 | 20.0 |
| **Health Board** | | | | | | |
| Ayrshire and Arran | 274848 | 7.4 | 227545 | 7.4 | 142557 | 7.1 |
| Borders | 78842 | 2.1 | 66156 | 2.2 | 40001 | 2.0 |
| Dumfries and Galloway | 108825 | 2.9 | 91196 | 3.0 | 54408 | 2.7 |
| Forth Valley | 203490 | 5.5 | 165237 | 5.4 | 108078 | 5.4 |
| Grampian | 408297 | 10.9 | 336417 | 11.0 | 225253 | 11.3 |
| Highland | 218699 | 5.9 | 181845 | 5.9 | 112743 | 5.6 |
| Lothian | 576535 | 15.5 | 471087 | 15.4 | 319087 | 15.9 |
| Orkney | 15059 | 0.4 | 12641 | 0.4 | 7803 | 0.4 |
| Shetland | 16065 | 0.4 | 13208 | 0.4 | 8559 | 0.4 |
| Western Isles | 18680 | 0.5 | 15658 | 0.5 | 9642 | 0.5 |
| Fife | 253631 | 6.8 | 209610 | 6.8 | 133706 | 6.7 |
| Tayside | 284924 | 7.6 | 234394 | 7.7 | 145869 | 7.3 |
| Greater Glasgow and Clyde | 807639 | 21.6 | 656602 | 21.5 | 438882 | 21.9 |
| Lanarkshire | 465303 | 12.5 | 378654 | 12.4 | 253968 | 12.7 |
| **Age** | | | | | | |
| 16-20 | 206797 | 5.5 | 0 | 0.0 | 0 | 0.0 |
| 21-25 | 256315 | 6.9 | 0 | 0.0 | 0 | 0.0 |
| 26-30 | 257011 | 6.9 | 49830 | 1.6 | 44237 | 2.2 |
| 31-35 | 257103 | 6.9 | 256933 | 8.4 | 241268 | 12.1 |
| 36-40 | 263673 | 7.1 | 263663 | 8.6 | 254730 | 12.7 |
| 41-45 | 253395 | 6.8 | 253361 | 8.3 | 245869 | 12.3 |
| 46-50 | 300310 | 8.0 | 300291 | 9.8 | 292611 | 14.6 |
| 51-55 | 343324 | 9.2 | 343298 | 11.2 | 335116 | 16.8 |
| 56-60 | 343786 | 9.2 | 343782 | 11.2 | 337009 | 16.8 |
| 61-65 | 310363 | 8.3 | 310349 | 10.1 | 249716 | 12.5 |
| >65 | 938760 | 25.2 | 938743 | 30.7 | 0 | 0.0 |
| **Sex** | | | | | | |
| Male | 1789964 | 48.0 | 1447656 | 47.3 | 969446 | 48.5 |
| Female | 1940873 | 52.0 | 1612594 | 52.7 | 1031110 | 51.5 |
| **Occupation** | | | | | | |
| Low risk | 1346460 | 36.1 | 1310187 | 42.8 | 904684 | 45.2 |
| Med risk | 1183541 | 31.7 | 1093595 | 35.7 | 707573 | 35.4 |
| High risk | 583180 | 15.6 | 573717 | 18.7 | 388299 | 19.4 |
| Economically inactive | 617656 | 16.6 | 82751 | 2.7 | NA | |
| **TOTAL** | 3730837 | 100.0 | 3060250 | 100.0 | 2000556 | 100.0 |

**Table S2:** Aggregation (categorical and binary) of 2011 Scottish Census ethnicity variable used in analysis.

| ***Dis-aggregated categories in 2011 Census*** | ***Categorical ethnicity variable*** | ***Binary ethnicity variable*** |
| --- | --- | --- |
| White Scottish | White Scottish | White |
| White Other British | White British or Irish |  |
| White Irish |  |  |
| White Gypsy/Traveller | Other White |  |
| White Polish |  |  |
| Other White |  |  |
| Pakistani, Pakistani Scottish or Pakistani British (referred to in main text as Pakistani) | South Asian | Non-White |
| Indian, Indian Scottish, or Indian British (referred to in main text as Indian) |  |  |
| Bangladeshi, Bangladeshi Scottish or Bangladeshi British (referred to in main text as Bangladeshi) |  |  |
| African, African Scottish, or African British (referred to in main text as African) | African, Caribbean, or Black |  |
| Caribbean, Caribbean Scottish or Caribbean British/Black, Black Scottish or Black British (referred to in main text as African or Black) |  |  |
| Arab, Arab Scottish, or Arab British (referred to in main text as Arab) | Other Ethnicity |  |
| Chinese, Chinese Scottish or Chinese British (referred to in main text as Chinese) |  |  |
| Other Asian |  |  |
| Mixed or Multiple Ethnicity |  |  |
| Other Ethnicity |  |  |

**Table S3:** List of occupations categorised into different COVID-19 risk levels.

| **3-digit SOC group** | **SOC Number** | **COVID-19 occupation risk** |
| --- | --- | --- |
| 111 111 'Chief Executives and Senior Officials' | 111 | Low |
| 112 112 'Production Managers and Directors' | 112 | Low |
| 113 113 'Functional Managers and Directors' | 113 | Low |
| 115 115 'Financial Institution Managers and Directors' | 115 | Low |
| 116 116 'Managers and Directors in Transport and Logistics' | 116 | Medium |
| 117 117 'Senior Officers in Protective Services' | 117 | Medium |
| 118 118 'Health and Social Services Managers and Directors' | 118 | Medium |
| 119 119 'Managers and Directors in Retail and Wholesale' | 119 | Medium |
| 121 121 'Managers and Proprietors in Agriculture Related Srvcs' | 121 | Low |
| 122 122 'Managers and Proprietors in Hsptlty and Leisure Srvcs' | 122 | Medium |
| 124 124 'Managers and Proprietors in Health and Care Services' | 124 | Medium |
| 125 125 'Managers and Proprietors in Other Services' | 125 | Medium |
| 211 211 'Natural and Social Science Professionals' | 211 | Low |
| 212 212 'Engineering Professionals' | 212 | Low |
| 213 213 'IT and Telecommunications Professionals' | 213 | Low |
| 214 214 'Conservation and Environment Professionals' | 214 | Low |
| 215 215 'Research and Development Managers ' | 215 | Low |
| 221 221 'Health Professionals' | 221 | High |
| 222 222 'Therapy Professionals' | 222 | High |
| 223 223 'Nursing and Midwifery Professionals' | 223 | High |
| 231 231 'Teaching and Educational Professionals' | 231 | High |
| 241 241 'Legal Professionals' | 241 | Low |
| 242 242 'Business, Research and Administrative Professionals' | 242 | Low |
| 243 243 'Architects, Town Planners and Surveyors' | 243 | Low |
| 244 244 'Welfare Professionals' | 244 | High |
| 245 245 'Librarians and Related Professionals' | 245 | Low |
| 246 246 'Quality and Regulatory Professionals' | 246 | Low |
| 247 247 'Media Professionals' | 247 | Low |
| 311 311 'Science, Engineering and Production Technicians' | 311 | Low |
| 312 312 'Draughtspersons and Related Architectural Technicians' | 312 | Low |
| 313 313 'Information Technology Technicians' | 313 | Low |
| 321 321 'Health Associate Professionals' | 321 | High |
| 323 323 'Welfare and Housing Associate Professionals' | 323 | High |
| 331 331 'Protective Service Occupations' | 331 | High |
| 341 341 'Artistic, Literary and Media Occupations' | 341 | Low |
| 342 342 'Design Occupations' | 342 | Low |
| 344 344 'Sports and Fitness Occupations' | 344 | Medium |
| 351 351 'Transport Associate Professionals' | 351 | Low |
| 352 352 'Legal Associate Professionals' | 352 | Low |
| 353 353 'Business, Finance and Related Associate Professionals' | 353 | Low |
| 354 354 'Sales, Marketing and Related Associate Professionals' | 354 | Low |
| 355 355 'Conservation and Environmental Assct Professionals' | 355 | Low |
| 356 356 'Public Services and Other Associate Professionals' | 356 | Low |
| 411 411 'Administrative Occupations: Gvrnmnt and Related Orgs' | 411 | Low |
| 412 412 'Administrative Occupations: Finance' | 412 | Low |
| 413 413 'Administrative Occupations: Records' | 413 | Low |
| 415 415 'Other Administrative Occupations' | 415 | Low |
| 416 416 'Administrative Occupations: Office Mngrs and Sprvsrs' | 416 | Low |
| 421 421 'Secretarial and Related Occupations' | 421 | Medium |
| 511 511 'Agricultural and Related Trades' | 511 | Low |
| 521 521 'Metal Forming, Welding and Related Trades' | 521 | Low |
| 522 522 'Metal Machining, Fitting and Instrument Making Trades' | 522 | Low |
| 523 523 'Vehicle Trades' | 523 | Low |
| 524 524 'Electrical and Electronic Trades' | 524 | Low |
| 525 525 'Skilled Metal, Electrical and Electronic Trds Sprvsrs' | 525 | Low |
| 531 531 'Construction and Building Trades' | 531 | Low |
| 532 532 'Building Finishing Trades' | 532 | Low |
| 533 533 'Construction and Building Trades Supervisors' | 533 | Low |
| 541 541 'Textiles and Garments Trades' | 541 | Low |
| 542 542 'Printing Trades' | 542 | Low |
| 543 543 'Food Preparation and Hospitality Trades' | 543 | Medium |
| 544 544 'Other Skilled Trades' | 544 | Low |
| 612 612 'Childcare and Related Personal Services' | 612 | High |
| 613 613 'Animal Care and Control Services' | 613 | Low |
| 614 614 'Caring Personal Services' | 614 | High |
| 621 621 'Leisure and Travel Services' | 621 | Medium |
| 622 622 'Hairdressers and Related Services' | 622 | Medium |
| 623 623 'Housekeeping and Related Services' | 623 | Medium |
| 624 624 'Cleaning and Housekeeping Managers and Supervisors' | 624 | Medium |
| 711 711 'Sales Assistants and Retail Cashiers' | 711 | Medium |
| 712 712 'Sales Related Occupations' | 712 | Medium |
| 713 713 'Sales Supervisors' | 713 | Medium |
| 721 721 'Customer Service Occupations' | 721 | Low |
| 722 722 'Customer Service Managers and Supervisors' | 722 | Low |
| 811 811 'Process Operatives' | 811 | Medium |
| 812 812 'Plant and Machine Operatives' | 812 | Low |
| 813 813 'Assemblers and Routine Operatives' | 813 | Low |
| 814 814 'Construction Operatives' | 814 | Low |
| 821 821 'Road Transport Drivers' | 821 | Medium |
| 822 822 'Mobile Machine Drivers and Operatives' | 822 | Low |
| 823 823 'Other Drivers and Transport Operatives' | 823 | Low |
| 911 911 'Elementary Agricultural Occupations' | 911 | Low |
| 912 912 'Elementary Construction Occupations' | 912 | Low |
| 913 913 'Elementary Process Plant Occupations' | 913 | Medium |
| 921 921 'Elementary Administration Occupations' | 921 | Medium |
| 923 923 'Elementary Cleaning Occupations' | 923 | Medium |
| 924 924 'Elementary Security Occupations' | 924 | Medium |
| 925 925 'Elementary Sales Occupations' | 925 | Medium |
| 926 926 'Elementary Storage Occupations' | 926 | Medium |
| 927 927 'Other Elementary Services Occupations' | 927 | Medium |

**Table S4:** Count of study population (aged ≥16 years) by ethnic group and Scottish Index of Multiple Deprivation (SIMD) level.

|  | **SIMD** | | | | | | | | | | |
| --- | --- | --- | --- | --- | --- | --- | --- | --- | --- | --- | --- |
| **Ethnic Group** | **1 (most deprived)** | | **2** | | **3** | | **4** | | **5 (least deprived)** | | **Total** |
|  | **n** | **%** | **n** | **%** | **n** | **%** | **n** | **%** | **n** | **%** | **n** |
| White Scottish | 646688 | 20.1 | 666910 | 20.7 | 648479 | 20.1 | 644263 | 20.0 | 617764 | 19.2 | 3224104 |
| White Other British & Irish | 29205 | 9.4 | 44168 | 14.3 | 74827 | 24.2 | 79694 | 25.7 | 81842 | 26.4 | 309736 |
| Other White | 21334 | 23.5 | 18914 | 20.8 | 16548 | 18.2 | 16219 | 17.8 | 17882 | 19.7 | 90897 |
| South Asian | 7823 | 16.0 | 8974 | 18.3 | 8171 | 16.7 | 11108 | 22.7 | 12874 | 26.3 | 48950 |
| African, Caribbean, or Black | 5361 | 36.4 | 2835 | 19.2 | 2102 | 14.3 | 2090 | 14.2 | 2341 | 15.9 | 14729 |
| Other | 7995 | 18.8 | 7163 | 16.9 | 7016 | 16.5 | 8521 | 20.1 | 11726 | 27.6 | 42421 |
| **TOTAL** | 718406 | 19.3 | 748964 | 20.1 | 757143 | 20.3 | 761895 | 20.4 | 744429 | 20.0 | 3730837 |
| White | 697227 | 19.2 | 729992 | 20.1 | 739854 | 20.4 | 740176 | 20.4 | 717488 | 19.8 | 3624737 |
| Non-white | 21179 | 20.0 | 18972 | 17.9 | 17289 | 16.3 | 21719 | 20.5 | 26941 | 25.4 | 106100 |
| **TOTAL** | 718406 | 19.3 | 748964 | 20.1 | 757143 | 20.3 | 761895 | 20.4 | 744429 | 20.0 | 3730837 |

**Table S5:** Count of cases of severe COVID-19 and positive SARS-CoV-2 tests by ethnic group and Scottish Index of Multiple Deprivation (SIMD) level.

| **Ethnic Group** | **Cases of severe COVID-19 (n) by SIMD** | | | | | **Positive SARS-CoV2 tests (n) by SIMD** | | | | |
| --- | --- | --- | --- | --- | --- | --- | --- | --- | --- | --- |
|  | 1 (most deprived) | 2 | 3 | 4 | 5 (least deprived) | 1 | 2 | 3 | 4 | 5 |
| White Scottish | 10322 | 8428 | 6498 | 5378 | 4494 | 177197 | 171375 | 149956 | 149925 | 143804 |
| White Other British & Irish | 444 | 468 | 514 | 500 | 443 | 6511 | 8568 | 11497 | 12971 | 14436 |
| Other White | 113 | 101 | 81 | 64 | 65 | 5766 | 5003 | 3760 | 3504 | 3474 |
| South Asian | 93 | 128 | 97 | 123 | 107 | 2205 | 2678 | 2394 | 3251 | 3505 |
| African, Caribbean, or Black | 43 | 18 | 15 | omitted as low case numbers | | 1443 | 786 | 523 | 502 | 547 |
| Other | 66 | 42 | 43 |  |  | 1946 | 1652 | 1437 | 1715 | 2300 |
| **TOTAL** | 10322 | 8428 | 6498 | 5378 | 4494 | 195068 | 190062 | 169567 | 171868 | 168066 |
| White | 10879 | 8997 | 7093 | 5942 | 5002 | 189474 | 184946 | 165213 | 166400 | 161714 |
| Non-white | 202 | 188 | 155 | 174 | 154 | 5594 | 5116 | 4354 | 5468 | 6352 |
| **TOTAL** | 11081 | 9185 | 7248 | 6116 | 5156 | 195068 | 190062 | 169567 | 171868 | 168066 |

**Table S6:** Count of study population (aged ≥30 years), cases of severe COVID-19, and positive SARS-CoV-2 tests by ethnic group and education level.

| **Ethnic group** | **Education level** | | | | | **Severe COVID-19 cases (n) by education level** | | **Positive SARS-CoV-2 tests (n) by education level** | |
| --- | --- | --- | --- | --- | --- | --- | --- | --- | --- |
|  | **Degree** | | **No degree** | | **Total** | **Degree** | **No degree** | **Degree** | **No degree** |
|  | **n** | **%** | **n** | **%** | **n** | **n** | **n** | **n** | **n** |
| White Scottish | 655001 | 25.0 | 1969351 | 75.0 | 2624352 | 4519 | 29699 | 135599 | 421811 |
| White Other British or Irish | 132658 | 47.9 | 144568 | 52.1 | 277226 | 691 | 1637 | 20958 | 23085 |
| Other White | 41505 | 52.8 | 37156 | 47.2 | 78661 | 136 | 277 | 8936 | 8767 |
| South Asian | 16181 | 44.1 | 20515 | 55.9 | 36696 | 135 | 381 | 4283 | 5574 |
| African, Caribbean, or Black | 6395 | 55.6 | 5107 | 44.4 | 11502 | 43 | 42 | 1487 | 1272 |
| Other Ethnicity | 15938 | 50.1 | 15875 | 49.9 | 31813 | 80 | 134 | 2974 | 2812 |
| **TOTAL** | 867678 | 28.4 | 2192572 | 71.6 | 3060250 | 5604 | 32170 | 174237 | 463321 |
| White | 829164 | 27.8 | 2151075 | 72.2 | 2980239 | 31613 | 5346 | 165493 | 453663 |
| Non-white | 38514 | 48.1 | 41497 | 51.9 | 80011 | 557 | 258 | 8744 | 9658 |
| **TOTAL** | 867678 | 28.4 | 2192572 | 71.6 | 3060250 | 32170 | 5604 | 174237 | 463321 |

**Table S7:** Count of study population (aged ≥30 & ≤64 years), cases of severe COVID-19, and positive SARS-CoV-2 tests by ethnic group and occupation risk. Note missing individuals were excluded from analyses and are not included in count of severe COVID-19 cases or SARS-CoV-2 tests

| **Ethnic Group** | **Occupational risk** | | | | | | | **Severe COVID-19 cases (n) by occupational risk** | | | **Positive SARS-CoV-2 tests (n) by occupational risk** | | |
| --- | --- | --- | --- | --- | --- | --- | --- | --- | --- | --- | --- | --- | --- |
|  | **Low** | | **Medium** | | **High** | | **Total** | **High** | **Med** | **Low** | **High** | **Med** | **Low** |
|  | **n** | **%** | **n** | **%** | n | % | **n** | **n** | **n** | **n** | **n** | **n** | **n** |
| White Scottish | 775142 | 45.6 | 597964 | 35.2 | 325120 | 19.14 | 1698226 | 100919 | 161496 | 191931 | 1626 | 3997 | 3432 |
| White Other British & Irish | 85199 | 48.6 | 49517 | 28.2 | 40610 | 23.16 | 175326 | 9580 | 10257 | 16061 | 115 | 204 | 256 |
| Other White | 24685 | 36.9 | 32577 | 48.6 | 9721 | 14.51 | 66983 | 2423 | 8900 | 5177 | 28 | 130 | 60 |
| South Asian | 8536 | 31.8 | 13353 | 49.8 | 4947 | 18.43 | 26836 | 1710 | 3667 | 2373 | 29 | 153 | 48 |
| African, Caribbean, or Black | 3362 | 35.5 | 3229 | 34.1 | 2886 | 30.45 | 9477 | 924 | 770 | 722 | 22 | 22 | 17 |
| Other Ethnicity | 7760 | 32.7 | 10933 | 46.1 | 5015 | 21.15 | 23708 | 1440 | 2046 | 1458 | 40 | 43 | 33 |
| **TOTAL** | 904684 | 45.2 | 707573 | 35.4 | 388299 | 19.41 | 2000556 | 116996 | 187136 | 217722 | 1860 | 4549 | 3846 |
| White | 885026 | 45.6 | 680058 | 35.0 | 375451 | 19.35 | 1940535 | 112922 | 180653 | 213169 | 1769 | 4331 | 3748 |
| Non-White | 19658 | 32.8 | 27515 | 45.8 | 12848 | 21.41 | 60021 | 4074 | 6483 | 4553 | 91 | 218 | 98 |
| **TOTAL** | 904684 | 45.2 | 707573 | 35.4 | 388299 | 19.41 | 2000556 | 116996 | 187136 | 217722 | 1860 | 4549 | 3846 |

**Table S8:** Percentage of missing values for education and occupation by ethnic group aged 30+ years

|  | **% missing** | |
| --- | --- | --- |
| ***Ethnic Group*** | **Education** | **Occupation** |
| White | 0.01 | 2.8 |
| Non-White | 0.02 | 14.1 |
| White Scottish | 0.0 | 2.8 |
| White Other British & Irish | 0.0 | 1.7 |
| Other White | 0.0 | 3.6 |
| South Asian | 0.0 | 15.2 |
| African, Caribbean, or Black | 0.0 | 10.6 |
| Other | 0.0 | 14.1 |
| TOTAL | 0.01 | 3.1 |

**Table S9:** Risk of severe COVID-19 – as hazard ratio (HR) with 95% confidence interval (CI) – within Scottish Index of Multiple Deprivation (SIMD) quintiles by ethnic group, with White group and White Scottish groups as reference for aggregated and disaggregated respectively; and within levels of ethnic group by SIMD quintile, with individuals in least deprived quintile (5) as reference group.

**Aggregated**

| **Severe COVID-19** | *SIMD* | | | | | | | | | |
| --- | --- | --- | --- | --- | --- | --- | --- | --- | --- | --- |
|  | 5 (least deprived) | | 4 | | 3 | | 2 | | 1 (most deprived) | |
| *Ethnic Group* | **HR** | **95% CI** | **HR** | **95% CI** | **HR** | **95% CI** | **HR** | **95% CI** | **HR** | **95% CI** |
| White | 1 | | 1 | | 1 | | 1 | | 1 | |
| Non-White | 1.427 | 1.215 , 1.676 | 1.646 | 1.415 , 1.915 | 1.643 | 1.401 , 1.927 | 1.505 | 1.302 , 1.739 | 1.153 | 1.003 , 1.325 |
| White | 1 | | 1.287 | 1.239 , 1.336 | 1.528 | 1.473 , 1.586 | 1.859 | 1.796 , 1.925 | 2.373 | 2.293 , 2.456 |
| Non-White | 1 | | 1.484 | 1.195 , 1.843 | 1.759 | 1.407 , 2.199 | 1.96 | 1.584 , 2.426 | 1.917 | 1.554 , 2.364 |

**Disaggregated**

| **Severe COVID-19** | *SIMD* | | | | | | | | | |
| --- | --- | --- | --- | --- | --- | --- | --- | --- | --- | --- |
|  | 5 (least deprived) | | 4 | | 3 | | 2 | | 1 (most deprived) | |
| *Ethnic Group* | **HR** | **95% CI** | **HR** | **95% CI** | **HR** | **95% CI** | **HR** | **95% CI** | **HR** | **95% CI** |
| White Scottish | 1 | | 1 | | 1 | | 1 | | 1 | |
| White British or Irish | 0.73 | 0.662 , 0.804 | 0.726 | 0.662 , 0.796 | 0.676 | 0.618 , 0.74 | 0.806 | 0.772 , 0.934 | 0.849 | 0.772 , 0.934 |
| Other White | 0.779 | 0.61 , 0.996 | 0.801 | 0.626 , 1.025 | 0.89 | 0.715 , 1.109 | 0.9 | 0.58 , 0.84 | 0.698 | 0.58 , 0.84 |
| South Asian | 1.79 | 1.477 , 2.168 | 2.066 | 1.728 , 2.472 | 1.959 | 1.603 , 2.395 | 2.011 | 1.075 , 1.618 | 1.319 | 1.075 , 1.618 |
| African, Caribbean, or Black | 1.013 | 0.506 , 2.027 | 1.196 | 0.643 , 2.224 | 1.482 | 0.893 , 2.46 | 1.141 | 0.828 , 1.508 | 1.117 | 0.828 , 1.508 |
| Other Ethnicity | 0.885 | 0.646 , 1.213 | 0.998 | 0.734 , 1.357 | 1.138 | 0.843 , 1.536 | 0.89 | 0.756 , 1.228 | 0.964 | 0.756 , 1.228 |
| White Scottish | 1 | | 1.281 | 1.231 , 1.333 | 1.522 | 1.465 , 1.582 | 1.819 | 1.754 , 1.887 | 2.325 | 2.243 , 2.41 |
| White British or Irish | 1 | | 1.275 | 1.121 , 1.449 | 1.411 | 1.242 , 1.603 | 2.009 | 1.764 , 2.288 | 2.706 | 2.371 , 3.087 |
| Other White | 1 | | 1.317 | 0.932 , 1.86 | 1.74 | 1.255 , 2.411 | 2.101 | 1.538 , 2.869 | 2.082 | 1.535 , 2.826 |
| South Asian | 1 | | 1.479 | 1.142 , 1.917 | 1.667 | 1.266 , 2.194 | 2.044 | 1.581 , 2.642 | 1.714 | 1.298 , 2.263 |
| African, Caribbean, or Black | 1 | | 1.512 | 0.597 , 3.831 | 2.228 | 0.944 , 5.254 | 2.048 | 0.891 , 4.711 | 2.564 | 1.205 , 5.454 |
| Other Ethnicity | 1 | | 1.445 | 0.932 , 2.24 | 1.958 | 1.269 , 3.02 | 1.83 | 1.184 , 2.83 | 2.532 | 1.704 , 3.762 |

**Table S10:** Risk of severe COVID-19 – as hazard ratio (HR) with 95% confidence interval (CI) – within levels of education by ethnic group, with White group and White Scottish as reference for aggregated and disaggregated groups respectively; and within levels of ethnic group by education, with degree group as reference.

**Aggregated**

| **Severe COVID-19** | Degree | | No Degree | |
| --- | --- | --- | --- | --- |
|  | **HR** | **95% CI** | **HR** | **95% CI** |
| White | 1 | | 1 | |
| Non-White | 1.745 | 1.54, 1.978 | 1.353 | 1.243, 1.472 |
| White | 1 | | 1.812 | 1.76, 1.866 |
| Non-White | 1 | | 1.404 | 1.212, 1.628 |

**Disaggregated**

| **Severe COVID-19** | Degree | | No Degree | |
| --- | --- | --- | --- | --- |
|  | **HR** | **95% CI** | **HR** | **95% CI** |
| White Scottish | 1 | | 1 | |
| White British or Irish | 0.782 | 0.722 , 0.847 | 0.761 | 0.724 , 0.8 |
| Other White | 0.86 | 0.725 , 1.021 | 0.942 | 0.837 , 1.061 |
| South Asian | 1.965 | 1.656 , 2.333 | 1.674 | 1.512 , 1.852 |
| African, Caribbean, or Black | 1.82 | 1.348 , 2.458 | 1.16 | 0.857 , 1.571 |
| Other Ethnicity | 1.31 | 1.05 , 1.634 | 0.872 | 0.736 , 1.033 |
| White Scottish | 1 | | 1.773 | 1.718 , 1.829 |
| White British or Irish | 1 | | 1.725 | 1.578 , 1.886 |
| Other White | 1 | | 1.942 | 1.581 , 2.384 |
| South Asian | 1 | | 1.51 | 1.24 , 1.837 |
| African, Caribbean, or Black | 1 | | 1.13 | 0.738 , 1.729 |
| Other Ethnicity | 1 | | 1.18 | 0.894 , 1.556 |

**Table S11:** Risk of severe COVID-19 – as hazard ratio (HR) with 95% confidence interval (CI) – within levels of occupational risk by ethnicity, with White group and White Scottish as reference for aggregated and disaggregated groups respectively and within levels of ethnic group by occupational risk, with individuals in low-risk occupations as reference.

**Aggregated**

| **Severe COVID-19** | *Occupational Risk* | | | | | |
| --- | --- | --- | --- | --- | --- | --- |
|  | Low risk | | Medium risk | | High risk | |
| *Ethnic Group* | **HR** | **95% CI** | **HR** | **95% CI** | **HR** | **95% CI** |
| White | 1 | | 1 | | 1 | |
| Non-White | 1.534 | 1.254 , 1.876 | 1.332 | 1.162 , 1.528 | 1.745 | 1.413 , 2.155 |
| White | 1 | | 1.556 | 1.488 , 1.626 | 1.11 | 1.047 , 1.178 |
| Non-White | 1 | | 1.351 | 1.065 , 1.715 | 1.263 | 0.949 , 1.681 |

**Disaggregated**

| **Severe COVID-19** |  |  |  |  |  |  |
| --- | --- | --- | --- | --- | --- | --- |
|  | Low | | Medium | | High | |
| *Ethnic Group* | **HR** | **95% CI** | **HR** | **95% CI** | **HR** | **95% CI** |
| White Scottish | 1 | | 1 | | 1 | |
| White British or Irish | 0.725 | 0.638 , 0.824 | 0.681 | 0.592 , 0.784 | 0.615 | 0.509 , 0.743 |
| Other White | 0.756 | 0.585 , 0.976 | 0.91 | 0.764 , 1.085 | 0.731 | 0.503 , 1.062 |
| South Asian | 1.682 | 1.264 , 2.238 | 1.78 | 1.514 , 2.094 | 1.353 | 0.937 , 1.953 |
| African, Caribbean, or Black | 1.548 | 0.961 , 2.494 | 1.305 | 0.858 , 1.985 | 1.806 | 1.185 , 2.751 |
| Other Ethnicity | 1.251 | 0.888 , 1.763 | 0.665 | 0.492 , 0.898 | 1.906 | 1.392 , 2.609 |
| White Scottish | 1 | | 1.548 | 1.478 , 1.621 | 1.128 | 1.061 , 1.199 |
| White British or Irish | 1 | | 1.454 | 1.209 , 1.748 | 0.956 | 0.767 , 1.192 |
| Other White | 1 | | 1.865 | 1.373 , 2.532 | 1.091 | 0.697 , 1.71 |
| South Asian | 1 | | 1.638 | 1.184 , 2.266 | 0.907 | 0.572 , 1.438 |
| African, Caribbean, or Black | 1 | | 1.305 | 0.693 , 2.457 | 1.316 | 0.699 , 2.478 |
| Other Ethnicity | 1 | | 0.823 | 0.523 , 1.295 | 1.719 | 1.084 , 2.725 |

**Table S12:** Risk of severe COVID-19 – as hazard ratio (HR) with 95% confidence interval (CI) – by ethnic group (binary) and occupational risk with White individuals in low-risk occupations as reference group, and by ethnic group (categorical) and occupational risk, with White Scottish individuals in low-risk occupations as reference group, across first wave of the COVID-19 pandemic.

| **Severe COVID-19 across WAVE 1** | *Occupational Risk* | | | | | |
| --- | --- | --- | --- | --- | --- | --- |
|  | **Low** | | **Medium** | | **High** | |
| *Ethnic Group (binary)* | **HR** | **95% CI** | **HR** | **95% CI** | **HR** | **95% CI** |
| White | 1 | | 1.631 | 1.42 , 1.873 | 1.566 | 1.324 , 1.851 |
| Non-White | 1.824 | 1 , 3.327 | 2.06 | 1.339 , 3.17 | 3.855 | 2.397 , 6.2 |
| Multiplicative interaction (ratio of HRs) |  |  | 0.692 | 0.335, 1.445 | 1.350 | 0.644, 2.754 |
| Additive interaction (RERI) |  |  | -0.395 | -1.92, 0.885 | 1.465 | -0.509, 3.826 |
| *Ethnic Group (categorical)* |  | | | | | |
| White Scottish | 1 | | 1.64 | 1.42 , 1.895 | 1.629 | 1.368 , 1.939 |
| White British or Irish | 0.86 | 0.595 , 1.243 | 1.449 | 0.978 , 2.147 | 0.859 | 0.502 , 1.469 |
| Other White | 0.542 | 0.202 , 1.453 | 0.629 | 0.26 , 1.522 | 0.928 | 0.298 , 2.896 |
| South Asian | 1.512 | 0.563 , 4.058 | 2.613 | 1.556 , 4.389 | 2.139 | 0.797 , 5.74 |
| African, Caribbean, or Black | 1.884 | 0.469 , 7.571 | 1.928 | 0.48 , 7.748 | 3.72 | 1.386 , 9.98 |
| Other Ethnicity | 2.004 | 0.828 , 4.85 | 1.206 | 0.499 , 2.917 | 5.443 | 2.898 , 10.224 |
| Multiplicative interaction (ratio of HRs) |  |  |  |  |  |  |
| White British or Irish |  |  | 1.027 | 0.616, 1.722 | 0.613 | 0.318, 1.144 |
| Other White |  |  | 0.708 | 0.203, 2.811 | 1.051 | 0.251, 4.684 |
| South Asian |  |  | 1.054 | 0.336, 3.273 | 0.868 | 0.231, 3.428 |
| African, Caribbean, or Black |  |  | 0.624 | 0.085, 4.706 | 1.212 | 0.238, 6.335 |
| Other Ethnicity |  |  | 0.367 | 0.106, 1.213 | 1.667 | 0.586, 4.806 |
| Additive interaction (RERI) |  |  |  |  |  |  |
| White British or Irish |  |  | -0.051 | -0.657, 0.646 | -0.63 | -1.208, 0.029 |
| Other White |  |  | -0.553 | -1.429, 0.465 | -0.243 | -1.269, 1.559 |
| South Asian |  |  | 0.461 | -2.285, 2.361 | -0.002 | -2.782, 3.616 |
| African, Caribbean, or Black |  |  | -0.596 | -6.461, 4.802 | 1.207 | -4.909, 7.812 |
| Other Ethnicity |  |  | -1.438 | -4.187, 0.537 | 2.810 | -0.734, 7.857 |

**Table S13:** Risk of severe COVID-19 – as hazard ratio (HR) with 95% confidence interval (CI) – by ethnic group (binary) and occupational risk with White individuals in low-risk occupations as reference group, and by ethnic group (categorical) and occupational risk, with White Scottish individuals in low-risk occupations as reference group, across second wave of the COVID-19 pandemic.

| **Severe COVID-19 across WAVE 2** | *Occupational Risk* | | | | | |
| --- | --- | --- | --- | --- | --- | --- |
|  | **Low** | | **Medium** | | **High** | |
| *Ethnic Group (binary)* | **HR** | **95% CI** | **HR** | **95% CI** | **HR** | **95% CI** |
| White | 1 | | 1.597 | 1.478 , 1.726 | 1.091 | 0.983 , 1.211 |
| Non-White | 1.905 | 1.366 , 2.657 | 2.502 | 2.004 , 3.125 | 3.192 | 2.374 , 4.29 |
| Multiplicative interaction (ratio of HRs) |  |  | 0.822 | 0.564, 1.23 | 1.536 | 0.987, 2.392 |
| Additive interaction (RERI) |  |  | 0.000 | -0.843, 0.788 | 1.196 | 0.139, 2.37 |
| *Ethnic Group (categorical)* |  |  |  |  |  |  |
| White Scottish | 1 | | 1.56 | 1.439 , 1.691 | 1.107 | 0.994 , 1.233 |
| White British or Irish | 0.609 | 0.476 , 0.78 | 1.127 | 0.88 , 1.443 | 0.57 | 0.394 , 0.825 |
| Other White | 0.655 | 0.393 , 1.09 | 1.632 | 1.197 , 2.224 | 0.701 | 0.333 , 1.474 |
| South Asian | 2.557 | 1.675 , 3.905 | 3.305 | 2.551 , 4.28 | 1.672 | 0.896 , 3.118 |
| African, Caribbean, or Black | 1.554 | 0.645 , 3.743 | 2.434 | 1.214 , 4.882 | 4.195 | 2.475 , 7.113 |
| Other Ethnicity | 1.144 | 0.594 , 2.206 | 1.142 | 0.686 , 1.901 | 3.867 | 2.533 , 5.901 |
| *Multiplicative interaction (ratio of HRs)* |  |  |  |  |  |  |
| White British or Irish |  |  | 1.186 | 0.851, 1.656 | 0.845 | 0.523, 1.306 |
| Other White |  |  | 1.597 | 0.909, 2.9 | 0.967 | 0.413, 2.43 |
| South Asian |  |  | 0.829 | 0.516, 1.378 | 0.591 | 0.281, 1.272 |
| African, Caribbean, or Black |  |  | 1.004 | 0.34, 3.12 | 2.439 | 0.97, 6.316 |
| Other Ethnicity |  |  | 0.640 | 0.286, 1.466 | 3.054 | 1.458, 6.865 |
| *Additive interaction (RERI)* |  |  |  |  |  |  |
| White British or Irish |  |  | -0.042 | -0.344, 0.283 | -0.146 | -0.437, 0.148 |
| Other White |  |  | 0.417 | -0.131, 1.03 | -0.061 | -0.582, 0.732 |
| South Asian |  |  | 0.188 | -1.29, 1.507 | -0.992 | -2.532, 0.738 |
| African, Caribbean, or Black |  |  | 0.32 | -1.864, 2.714 | 2.534 | 0.112, 5.66 |
| Other Ethnicity |  |  | -0.562 | -1.733, 0.338 | 2.616 | 1.027, 4.598 |

**Table S14:** Risk of severe COVID-19 – as hazard ratio (HR) with 95% confidence interval (CI) – by ethnic group (binary) and occupational risk with White individuals in low-risk occupations as reference group, and by ethnic group (categorical) and occupational risk, with White Scottish individuals in low-risk occupations as reference group, across third wave of the COVID-19 pandemic.

| **Severe COVID-19 across WAVE 3** | *Occupational Risk* | | | | | |
| --- | --- | --- | --- | --- | --- | --- |
|  | **Low** | | **Medium** | | **High** | |
| *Ethnic Group (binary)* | **HR** | **95% CI** | **HR** | **95% CI** | **HR** | **95% CI** |
| White | 1 | | 1.53 | 1.411 , 1.66 | 1.056 | 0.947 , 1.177 |
| Non-White | 1.598 | 1.129 , 2.262 | 2.48 | 1.975 , 3.114 | 1.569 | 1.038 , 2.374 |
| Multiplicative interaction (ratio of HRs) |  |  | 1.014 | 0.674, 1.499 | 0.930 | 0.544, 1.555 |
| Additive interaction (RERI) |  |  | 0.352 | -0.452, 1.082 | -0.085 | -0.934, 0.832 |
| *Ethnic Group (categorical)* |  | | | | | |
| White Scottish | 1 | | 1.525 | 1.401 , 1.66 | 1.069 | 0.953 , 1.197 |
| White British or Irish | 0.713 | 0.565 , 0.899 | 1.003 | 0.773 , 1.303 | 0.69 | 0.492 , 0.967 |
| Other White | 1.04 | 0.709 , 1.526 | 1.588 | 1.19 , 2.12 | 1.023 | 0.564 , 1.855 |
| South Asian | 1.423 | 0.823 , 2.463 | 3.361 | 2.565 , 4.404 | 2.388 | 1.408 , 4.05 |
| African, Caribbean, or Black | 1.96 | 0.932 , 4.125 | 2.589 | 1.342 , 4.993 | 1.193 | 0.447 , 3.187 |
| Other Ethnicity | 1.542 | 0.892 , 2.666 | 1.167 | 0.701 , 1.944 | 0.862 | 0.358 , 2.075 |
| *Multiplicative interaction (ratio of HRs)* |  |  |  |  |  |  |
| White British or Irish |  |  | 0.922 | 0.653, 1.282 | 0.905 | 0.603, 1.344 |
| Other White |  |  | 1.001 | 0.641, 1.543 | 0.920 | 0.458, 1.891 |
| South Asian |  |  | 1.549 | 0.831, 2.833 | 1.570 | 0.743, 3.323 |
| African, Caribbean, or Black |  |  | 0.866 | 0.313, 2.463 | 0.569 | 0.181, 1.97 |
| Other Ethnicity |  |  | 0.496 | 0.242, 1.012 | 0.523 | 0.182, 1.599 |
| *Additive interaction (RERI)* |  |  |  |  |  |  |
| White British or Irish |  |  | -0.235 | -0.558, 0.086 | -0.092 | -0.380, 0.228 |
| Other White |  |  | 0.023 | -0.56, 0.601 | -0.086 | -0.749, 0.840 |
| South Asian |  |  | 1.413 | 0.087, 2.629 | 0.896 | -0.577, 2.562 |
| African, Caribbean, or Black |  |  | 0.104 | -2.413, 2.702 | -0.836 | -3.073, 1.387 |
| Other Ethnicity |  |  | -0.900 | -2.104, 0.062 | -0.749 | -1.975, 0.615 |

**Table S15:** Risk of severe COVID-19 – as hazard ratio (HR) with 95% confidence interval (CI) – by ethnic group (binary) and occupational risk with White individuals in low-risk occupations as reference group, and by ethnic group (categorical) and occupational risk, with White Scottish individuals in low-risk occupations as reference group, across fourth wave of the COVID-19 pandemic.

| **Hosp & death WAVE 4** | *Occupational Risk* | | | | | |
| --- | --- | --- | --- | --- | --- | --- |
|  | **Low** | | **Medium** | | **High** | |
| *Ethnic Group (binary)* | **HR** | **95% CI** | **HR** | **95% CI** | **HR** | **95% CI** |
| White | 1 | | 1.509 | 1.389 , 1.639 | 1.016 | 0.909 , 1.136 |
| Non-White | 0.97 | 0.616 , 1.529 | 1.105 | 0.785 , 1.555 | 0.352 | 0.146 , 0.847 |
| Multiplicative interaction (ratio of HRs) |  |  | 0.755 | 0.426, 1.282 | -0.374 | -1.021, 0.179 |
| Additive interaction (RERI) |  |  | 0.357 | 0.128, 0.924 | -0.634 | -1.251, -0.063 |
| *Ethnic Group (categorical)* |  | | | | | |
| White Scottish | 1 | | 1.529 | 1.402 , 1.667 | 1.032 | 0.919 , 1.16 |
| White British or Irish | 0.822 | 0.66 , 1.024 | 0.925 | 0.704 , 1.215 | 0.725 | 0.522 , 1.008 |
| Other White | 0.641 | 0.391 , 1.052 | 1.161 | 0.827 , 1.63 | 0.654 | 0.31 , 1.375 |
| South Asian | 1.159 | 0.621 , 2.164 | 1.459 | 0.964 , 2.208 | 0.178 | 0.025 , 1.264 |
| African, Caribbean, or Black | 0.888 | 0.286 , 2.758 | 0.905 | 0.291 , 2.812 | 0.306 | 0.043 , 2.172 |
| Other Ethnicity | 0.738 | 0.331 , 1.648 | 0.643 | 0.32 , 1.289 | 0.526 | 0.169 , 1.633 |
| Multiplicative interaction (ratio of HRs) |  |  |  |  |  |  |
| White British or Irish |  |  | 0.736 | 0.517, 1.02 | -0.426 | -0.73, -0.116 |
| Other White |  |  | 1.185 | 0.674, 2.145 | 0.595 | -0.494, 0.512 |
| South Asian |  |  | 0.823 | 0.376, 1.768 | 0.139 | -1.388, 0.749 |
| African, Caribbean, or Black |  |  | 0.667 | 0.124, 3.287 | -0.442 | -2.593, 1.452 |
| Other Ethnicity |  |  | 0.570 | 0.221, 1.646 | 0.000 | -1.504, 0.135 |
| Additive interaction (RERI) |  |  |  |  |  |  |
| White British or Irish |  |  | 0.855 | 0.576, 1.254 | -0.129 | -0.434, 0.195 |
| Other White |  |  | 0.989 | 0.419, 2.247 | -0.019 | -0.53, 0.67 |
| South Asian |  |  | 0.149 | 0.021, 1.166 | -1.013 | -2.024, 0.109 |
| African, Caribbean, or Black |  |  | 0.334 | 0.032, 3.168 | -0.614 | -2.45, 1.146 |
| Other Ethnicity |  |  | 0.691 | 0.161, 2.905 | -0.244 | -1.206, 0.971 |
