# Supplementary material for: Effect modification and interaction between ethnicity and socioeconomic factors in severe COVID-19: analyses of linked national data for Scotland
Source: BMJ Open. 2025 Apr 14;15(4):e092727. doi: 10.1136/bmjopen-2024-092727 (PMC11997826; doi:10.1136/bmjopen-2024-092727)
Supplement: Supplementary file 2 [file bmjopen-2024-092727.reviewer_comments.pdf]

PEER REVIEW HISTORY

BMJ Open publishes all reviews undertaken for accepted manuscripts. Reviewers are asked to complete a checklist review form and are provided with free text boxes to elaborate on their assessment. These free text comments are reproduced below.

ARTICLE DETAILS

Title (Provisional)

Effect modification and interaction between ethnicity and socio-economic factors in severe COVID-19: analyses of linked national data for Scotland

Authors

McCabe, Ronan; Kibuchi, Eliud; Amele, Sarah; Irizar , Patricia; Sheikh, Aziz; Jeffrey, Karen; Ruden, Igor; Simpson, Colin R; McCowan, Colin; Ritchie, Lewis; Robertson, Chris; Leyland, Alastair H.; Demou, Evangelia; Pearce, Anna; Katikireddi, Srinivasa Vittal

---

VERSION 1 - REVIEW

**Reviewer**	**1**
**Name**	**Huang, Andrew**
**Affiliation**	**Brown University**
**Date**	**09-Oct-2024**
**COI**	**None**

General comments:

In this paper, the authors use observational data to examine the effects of (1) socioeconomic area deprivation, (2) education level, and (3) occupational risk with severe COVID-19 outcomes in Scotland, with specific interest in whether being in a minority ethnic group resulted in additive or multiplicative differences in these effects. Scottish health records and national registry data are used to identify the COVID-19 outcomes from March 1, 2020 to April 30, 2022 with the linked 2011 Scottish Census used to identify the exposure variables.

Overall, this paper addresses an important question regarding potential racial and ethnic health disparities during the COVID-19 pandemic in Scotland, and does well to explain the importance of analyses that can examine effect modification by race and ethnicity to better understand the impact of interpersonal, institutional, and structural racism on health outcomes in Scotland. Strengths of this study include the large size of the study population and the use of health records and national registries to identify severe COVID-19 outcomes during the follow-up period. When comparing Non-White residents to White residents, the study finds evidence of effect modification by ethnicity for socioeconomic area deprivation (quintiles 3 and 4 vs. least deprived 5) and occupational risk (high vs. low), but not for education degree attainment.

However, there are several major concerns with the study that limit the interpretation of its findings. First, while the use of 2011 Census data to identify self-reported ethnicity is valuable, I am skeptical that it can be a viable source of data for residence (used for socioeconomic area deprivation) or occupation (used for occupational risk) at the start of the COVID-19 follow-up period 9 years later, unless I am missing evidence that residence or occupation did not change systematically between ethnic groups between 2011 and 2020. And even if the residence or occupational status were valid at the baseline of the follow-up period, there would be residual time-varying confounding during the follow-up period due to changes in residence or occupation that may have occurred during the COVID-19 pandemic and occurred disproportionately among ethnic groups. In other words, it is easy to imagine that a change in occupational status (e.g., unemployment or a shift from low to high risk occupation) is associated with both ethnicity and severe COVID-19 outcomes. There is a secondary analysis presented that stratifies results by pandemic wave, but there needs to be significantly more discussion on the results of this secondary analysis and how it addresses the above concern or impacts the study results.

Second, it is unclear to me why receipt of COVID-19 vaccination and/or COVID-19 treatment (e.g., antibody) is not included as a confounder or an outcome of interest in these analyses, as it appears to be available in the data source and there are several studies in the literature indicating that vaccination is associated with both ethnicity and severe COVID-19 outcomes in the UK (Hussain B, Latif A, Timmons S, Nkhoma K, Nellums LB. Overcoming COVID-19 vaccine hesitancy among ethnic minorities: A systematic review of UK studies. Vaccine. 2022;40(25):3413-3432. doi:10.1016/j.vaccine.2022.04.030; Kamal A, Hodson A, Pearce JM. A Rapid Systematic Review of Factors Influencing COVID-19 Vaccination Uptake in Minority Ethnic Groups in the UK. Vaccines (Basel). 2021;9(10):1121. Published 2021 Oct 1. doi:10.3390/vaccines9101121; Woolf K, McManus IC, Martin CA, et al. Ethnic differences in SARS-CoV-2 vaccine hesitancy in United Kingdom healthcare workers: Results from the UK-REACH prospective nationwide cohort study. Lancet Reg Health Eur. 2021;9:100180. doi:10.1016/j.lanepe.2021.100180). If the goal of the study is to examine the impact of interpersonal, institutional, and structural racism on COVID-19 outcomes in Scotland, then the study would be strengthened by including an analysis of these effects on vaccination or treatment, as disparate access to care could explain or exacerbate the modification of ethnicity on the effect of socioeconomic status, education, or occupation on severe COVID-19 outcomes.

I applaud the authors for their work on this study and suggest several revisions to consider that may strengthen the study in revision. Additional specific comments are included in the attachment and below.

Specific comments:

1. The sections on Population, Exposure, Effect modifiers and Model confounders could be written more clearly to describe that there are three different analyses being presented.
    a. For example, in lines 36-40, only socioeconomic area deprivation and educational level are mentioned as effect modifiers, but not occupational risk. But occupational risk is presented as the third analysis everywhere else in the paper.

    b. Because there are three different study populations and three different analyses in this study, I am more inclined to define (1) socioeconomic area deprivation, (2) education level, and (3) occupational risk as the three exposures of interest and I would move ethnicity to the effect modifier section as the effect modifier of interest.
    c. A directed acyclic diagram (DAG) or series of DAGs would be helpful for visualizing the three analyses presented in this paper.
2. In Exposure, additional description of the Scottish Index of Multiple Deprivation would be appreciated for those unfamiliar with how the index is generated.
3. In Exposure, additional description of the occupational risk categories would be appreciated for those unfamiliar with how the low, middle and high risk categories are defined.
4. In Population, how was residency at the start of follow-up (March 1 2020) established? From what data source?
5. In model confounders, it is not geography as stated in the abstract but “health board (regional authorities responsible for health service delivery)” that is included as an explicit confounder in the analyses. Please explain this discrepancy - are these two different variables? As I understand it, including one but not the other would potentially address different baseline confounders.
6. In statistical analyses, please provide either statistical tests and/or graphical diagnostics for the assumption of proportional hazards to justify the use of the Cox model and the reporting of a constant hazard ratio over time.
7. In statistical analyses, for the secondary analyses, it is unclear to me why these specific time periods were chosen as pandemic waves to stratify the analysis. Are they associated with changes in prevalence? Changes in dominant COVID-19 strains? Or presumed economic and occupational changes?
8. In Table 1 and Table 2, please provide a footnote or visual clarity that clearly states that the hazard ratios being reported are from three different models fit on three different study populations. As of now, it may be mistakenly assumed that all three exposures are coefficients from the same model.
9. In Appendix 1 Figure A, please be more specific about the age cutoffs. Are these the ages of study participants at the time of the 2011 Census or at the time of follow-up start (March 1 2020)? Please specify this more clearly in the table notes.
10. In Appendix 1 Figure A, it appears that there are 1,339,555 individuals missing ethnicity data that were excluded from all 3 study populations, which is over 25% of the original population in the register. Please include this exclusion in the main paper in the population section. What can you tell me about these individuals? Can you discuss how this exclusion might affect the generalizability of your results to the general Scottish population?
11. In Appendix 1 Table A, please include a note or visual representation that specifies whether the columns represent the ages of study participants at the time of the 2011 Census or at the time of follow-up start on March 1 2020.
    a. The row for “Missing” under “Education level” appears to be missing for the last two age categories
    b. The row for “Missing” under “Occupation” appears to be missing for the last two age categories
12. In Appendix 1 Table B, it appears that Missingness is not random and that individuals from minority ethnic groups are more likely to be missing education and occupation

information. Please discuss this in more detail in the main paper – how you address missingness in the study population section, and the prevalence of missingness in the results section, and how this missingness affects your results in the discussion section.

13. In Appendix 7 Tables A-D, please provide a reference to the dates of the time period defined for each wave in the footnotes or visually in the table

---

**Reviewer**	**2**
**Name**	**Mateo-Urdiales, Alberto**
**Affiliation Department**	**Istituto Superiore di Sanità, Infectious Diseases**
**Date**	**09-Jan-2025**
**COI**	**None**

Thank you for this interesting paper which aims to answer an important public health question.

I think the paper is well written, the methods rigorous and clearly explained; and that the results are well presented. I only have minor comments:

Methods:

- You have used a definition of COVID-19 hospitalisation and COVID-19 death which included any hospitalisation/death after a positive test. The specificity of this definition may have varied during the pandemic, meaning that in some periods it may include many admissions/deaths that in reality were caused by something different than COVID-19. This misclassification may be differential according to exposure (ethnicity and deprivation) because those most vulnerable (e.g., most deprived) may be more likely to end up in hospital for reasons other than COVID. If you agree, I'd include a sentence in the limitations about this.

Discussion:

- I miss in the discussion is to describe the results in the context of previous studies. You mention in the introduction why ethnical inequalities in COVID may be present; and why socioeconomic inequalities may be present, but in the discussion the reader may want to know whether these results agree or disagree with previous studies and why that may be.

That's all. Thanks for the paper.

VERSION 1 - AUTHOR RESPONSE

Reviewer 1
General comments:

In this paper, the authors use observational data to examine the effects of (1) socioeconomic area deprivation, (2) education level, and (3) occupational risk with severe COVID-19 outcomes in Scotland, with specific interest in whether being in a minority ethnic group resulted in additive or multiplicative differences in these effects. Scottish health records and national registry data are used to identify the COVID-19 outcomes from March 1, 2020 to April 30, 2022 with the linked 2011 Scottish Census used to identify the exposure variables.

R1.1: Overall, this paper addresses an important question regarding potential racial and ethnic health disparities during the COVID-19 pandemic in Scotland and does well to explain the importance of analyses that can examine effect modification by race and ethnicity to better understand the impact of interpersonal, institutional, and structural racism on health outcomes in Scotland. Strengths of this study include the large size of the study population and the use of health records and national registries to identify severe COVID-19 outcomes during the follow-up period. When comparing Non-White residents to White residents, the study finds evidence of effect modification by ethnicity for socioeconomic area deprivation (quintiles 3 and 4 vs. least deprived 5) and occupational risk (high vs. low), but not for education degree attainment. However, there are several major concerns with the study that limit the interpretation of its findings. First, while the use of 2011 Census data to identify self-reported ethnicity is valuable, I am skeptical that it can be a viable source of data for residence (used for socioeconomic area deprivation) or occupation (used for occupational risk) at the start of the COVID-19 follow-up period 9 years later, unless I am missing evidence that residence or occupation did not change systematically between ethnic groups between 2011 and 2020. And even if the residence or occupational status were valid at the baseline of the follow-up period, there would be residual time-varying confounding during the follow-up period due to changes in residence or occupation that may have occurred during the COVID-19 pandemic and occurred disproportionately among ethnic groups. In other words, it is easy to imagine that a change in occupational status (e.g., unemployment or a shift from low to high risk occupation) is associated with both ethnicity and severe COVID-19 outcomes. There is a secondary analysis presented that stratifies results by pandemic wave, but there needs to be significantly more discussion on the results of this secondary analysis and how it addresses the above concern or impacts the study results.

A: We agree use of Census 2011to identify education and occupation may be susceptible to biased estimates. However, newer data are still not available. We have included the following in the discussion:

"It is worth noting that data on ethnicity, education, and occupation were captured in the 2011 Census and may have changed by the time of study. As such, this may have resulted in biased estimates. However, data for area deprivation were based on the CHI register (which is continually updated) rather than the 2011 Census and therefore has less potential for bias arising from misclassification."

R1.2: Second, it is unclear to me why receipt of COVID-19 vaccination and/or COVID-19 treatment (e.g., antibody) is not included as a confounder or an outcome of interest in these

analyses, as it appears to be available in the data source and there are several studies in the literature
indicating that vaccination is associated with both ethnicity and severe COVID-19 outcomes in the UK (Hussain B, Latif A, Timmons S, Nkhoma K, Nellums LB. Overcoming COVID-19 vaccine hesitancy among ethnic minorities: A systematic review of UK studies. Vaccine. 2022;40(25):3413-3432. doi:10.1016/j.vaccine.2022.04.030; Kamal A, Hodson A, Pearce JM. A Rapid Systematic Review of Factors Influencing COVID-19 Vaccination Uptake in Minority Ethnic Groups in the UK. Vaccines (Basel). 2021;9(10):1121. Published 2021 Oct 1. doi:10.3390/vaccines9101121; Woolf K, McManus IC, Martin CA, et al. Ethnic differences in SARS-CoV-2 vaccine hesitancy in United Kingdom healthcare workers: Results from the UKREACH prospective nationwide cohort study. Lancet Reg Health Eur. 2021;9:100180. doi:10.1016/j.lanepe.2021.100180). If the goal of the study is to examine the impact of interpersonal, institutional, and structural racism on COVID-19 outcomes in Scotland, then the study would be strengthened by including an analysis of these effects on vaccination or treatment, as disparate access to care could explain or exacerbate the modification of ethnicity on the effect of socioeconomic status, education, or occupation on severe COVID-19 outcomes.

A: As consistent with our pre-specified Directed Acyclic Graph (DAG), we consider vaccination and COVID-19 treatment as mediators of ethnic inequalities. This is consistent with the perspective of the UK Scientific Advisory Group on Emergencies subgroup on ethnicity which also considered these variables as mediators. While we agree that studying the potential for mediation through differential treatment and differential vaccination would be of interest, that would be a different research question from that which we addressed. We do however now note that this would be a fruitful avenue for potential further research:

R1.3 I applaud the authors for their work on this study and suggest several revisions to consider that may strengthen the study in revision. Additional specific comments are included in the attachment and below.

A: We are pleased for your comments and suggestions.

Specific comments:

1. The sections on Population, Exposure, Effect modifiers and Model confounders could be written more clearly to describe that there are three different analyses being presented.
    a. For example, in lines 36-40, only socioeconomic area deprivation and educational level are mentioned as effect modifiers, but not occupational risk. But occupational risk is presented as the third analysis everywhere else in the paper.

       A: We apologise that the wording was not as clear as it could have been. Occupation was mentioned in the ‘Effect modifiers’ section, with two measures of socio-economic disadvantage (SIMD and education) mentioned

and occupational risk. We have now updated the wording to make this clearer.

b. Because there are three different study populations and three different analyses in this study, I am more inclined to define (1) socioeconomic area deprivation, (2) education level, and (3) occupational risk as the three exposures of interest and I would move ethnicity to the effect modifier section as the effect modifier of interest.

A: We appreciate the reviewer's perspective that the suggested change would potentially be easier to follow. However, the interpretation would be subtly altered and would therefore be somewhat different to our pre-planned analysis. In particular, we note the distinction previously made about how to define an effect modifier which we have followed. Changing what is being characterised as the effect modifier could result in changes to the variables which would be used for statistical adjustment and we therefore feel would not be warranted post-hoc.

2.

a. A directed acyclic diagram (DAG) or series of DAGs would be helpful for visualizing the three analyses presented in this paper.

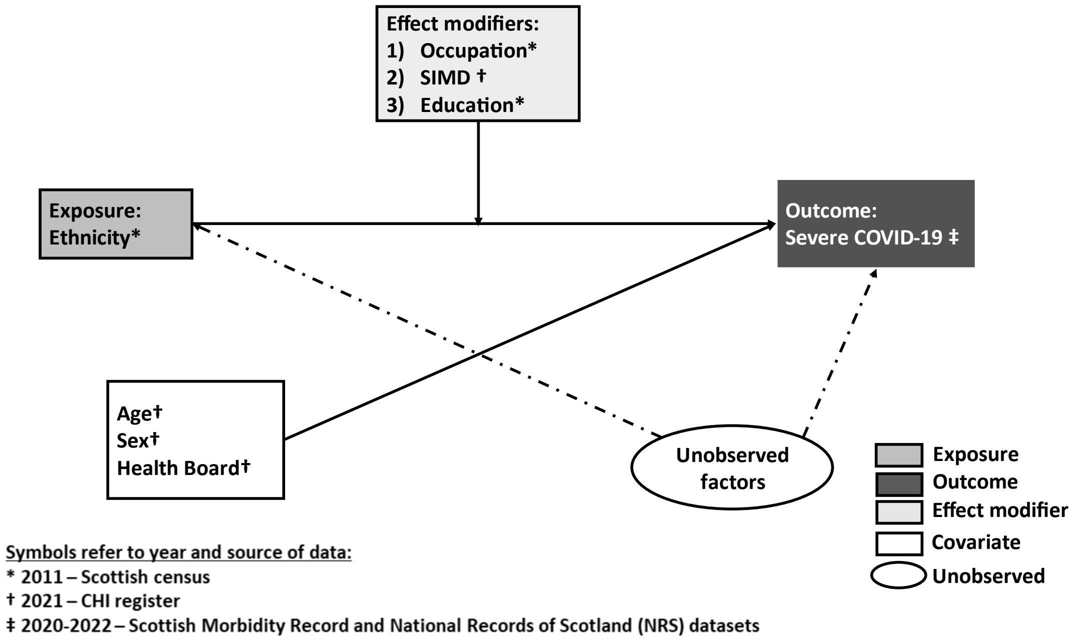

Figure 1: Directed acyclic graph (DAG) representing the relationship between COVID-19, ethnicity, effect modifiers (occupation, SIMD, education), and confounders (age, sex and health board). Each effect modifier was explored in a separate model.

3. In Exposure, additional description of the Scottish Index of Multiple Deprivation would be appreciated for those unfamiliar with how the index is generated.
   A: More details on SIMD have been added to the effect modifier section

“Area deprivation was measured using the 2020 Scottish Index of Multiple Deprivation (SIMD) data obtained from the CHI register. SIMD ranks small ‘data zones’ in Scotland (composed of ~700 individuals) by level of deprivation measured across seven weighted domains (employment, health, education, housing, income, service access, and crime across the region). We used SIMD quintiles, with quintile 1 indicating the most deprived 20% of data zones and quintile 5 the least deprived 20% data zones. While health is one of the domains within SIMD, it makes a small contribution to the overall index and previous work has shown that this results in minimal bias.”

4. In Exposure, additional description of the occupational risk categories would be appreciated for those unfamiliar with how the low, middle and high risk categories are defined.
   A: More details on occupation have been added to the effect modifier section
   “We classified occupation into three categories based on by likelihood of SARS-CoV-2 exposure (low-, medium-, and high-risk). These categories were adapted from previously published classifications[20] by five authors (EK, RM, AP, ED, and SVK) who independently assessed infection and mortality risks using three-digit Standardised Occupational Classification (SOC 2010) codes. Where there was a discrepancy, authors discussed and reached a consensus on the appropriate level of risk see Supplementary Table 2) “

5. In Population, how was residency at the start of follow-up (March 1 2020) established? From what data source?
   A:This is determined based on data from the CHI register, text has been updated to make clearer.

6. In model confounders, it is not geography as stated in the abstract but “health board (regional authorities responsible for health service delivery)” that is included as an explicit confounder in the analyses. Please explain this discrepancy - are these two different variables? As I understand it, including one but not the other would potentially address different baseline confounders.
   A: When geography was mentioned in the abstract it was referring to health board. This has been updated to improve clarity. *“We used electronic health records linked to the 2011 Census and Cox proportional hazards models, adjusting for age, sex, and health board.”*

7. In statistical analyses, please provide either statistical tests and/or graphical diagnostics for the assumption of proportional hazards to justify the use of the Cox model and the reporting of a constant hazard ratio over time.
   A: We used Cox regression because the time for the first hospitalisation was the outcome of interest and all assumptions to fit the model were met. We have extended the sentence in the “Statistical analyses” section to read *“ We used Cox proportional hazard models to estimate the risk of severe COVID-19 since the time for the first COVID-19 hospitalisation or death was the outcome of interest .”*

8. In statistical analyses, for the secondary analyses, it is unclear to me why these specific time periods were chosen as pandemic waves to stratify the analysis. Are they associated with changes in prevalence? Changes in dominant COVID-19 strains? Or presumed economic and occupational changes?
   A: *The waves were primarily defined based on reproduction rate, the growth rate and the positivity rate. However, there was no strict definition of when the wave starts or ends. Wave 3 was driven by the Delta variant while during wave 4 the Omicron variant was more prevalent. Added text to manuscript to make this clear*
   "These were defined following previous studies as wave 1 (1 March 2020 to 31 July 2020), wave 2 (1 August 2020 to 30 April 2021), wave 3 (1 May 2021 to 17 December 2021), and wave 4 (18 December 2021 to 30 April 2022) which were informed by changes in reproduction, growth, and positivity rates."

9. In Table 1 and Table 2, please provide a footnote or visual clarity that clearly states that the hazard ratios being reported are from three different models fit on three different study populations. As of now, it may be mistakenly assumed that all three exposures are coefficients from the same model.
   A: The footnotes of Table 1 and Table 2 have been updated to make it clearer that results are from 3 different models *"Results are based on three separate models on three different study populations exploring each effect modifier separately".*

10. In Appendix 1 Figure A, please be more specific about the age cutoffs. Are these the ages of study participants at the time of the 2011 Census or at the time of follow-up start (March 1 2020)? Please specify this more clearly in the table notes.
    A: The ages refer to age at the time of follow-up (March 1st, 2020). This has been made clearer in Appendix 1 Figure A

11. In Appendix 1 Figure A, it appears that there are 1,339,555 individuals missing ethnicity data that were excluded from all 3 study populations, which is over 25% of the original population in the register. Please include this exclusion in the main paper in the population section. What can you tell me about these individuals? Can you discuss how this exclusion might affect the generalizability of your results to the general Scottish population?
    A: Sentence about the number of individuals missing ethnicity data has been added to the beginning of the results section. *"There were 5,121,530 individuals in the EAVE-11 dataset, 51,138 of which did not have data on health board and 1, 139,555 who did not have disaggregated ethnicity data and were excluded from the analysis."*

12. In Appendix 1 Table A, please include a note or visual representation that specifies whether the columns represent the ages of study participants at the time of the 2011 Census or at the time of follow-up start on March 1 2020.
    A: Updated to include footnote specifying age refers to age at the start of the follow-up period. We have also updated in Data section *"Age (as a restricted cubic spline with four degrees of freedom) as of March 2020, sex, and health board (regional authorities responsible for health service delivery) were pre-specified confounders for all analyses."*

a. The row for "Missing" under "Education level" appears to be missing for the last two age categories
   A: Everyone aged 30+ had education data available therefore there was no missing data for the last two age categories.
b. The row for "Missing" under "Occupation" appears to be missing for the last two age categories
   A: There is no missing data for the 16+ and 30+ age groups since we only considered individuals in active occupations and that is the reason we have indicated NA. Everyone aged 30-64 had occupation data available therefore there was no missing data for that age categories.

13. In Appendix 1 Table B, it appears that Missingness is not random and that individuals from minority ethnic groups are more likely to be missing education and occupation information. Please discuss this in more detail in the main paper – how you address missingness in the study population section, and the prevalence of missingness in the results section, and how this missingness affects your results in the discussion section.
    A: We have included this as one of the study limitations. Moreover, missingness of more than 5% in the occupational risk among minority ethnic may have resulted to biased estimates

14. In Appendix 7 Tables A-D, please provide a reference to the dates of the time period defined for each wave in the footnotes or visually in the table
    A: We have included the following sentence in footnote *"Wave 1 (1 March 2020 to 31 July 2020), wave 2 (1 August 2020 to 30 April 2021), wave 3 (1 May 2021 to 17 December 2021), and wave 4 (18 December 2021 to 30 April 2022)."*

Reviewer: 2
Dr. Alberto Mateo-Urdiales, Istituto Superiore di Sanità
Comments to the Author:
Thank you for this interesting paper which aims to answer an important public health question.
I think the paper is well written, the methods rigorous and clearly explained; and that the results are well presented. I only have minor comments:

Methods:

R2.1- You have used a definition of COVID-19 hospitalisation and COVID-19 death which included any hospitalisation/death after a positive test. The specificity of this definition may have varied during the pandemic, meaning that in some periods it may include many admissions/deaths that in reality were caused by something different than COVID-19. This misclassification may be differential according to exposure (ethnicity and deprivation) because those most vulnerable (e.g., most deprived) may be more likely to end up in hospital for reasons other than COVID. If you agree, I'd include a sentence in the limitations about this.

A: We have added the following text to the discussion in response:

"While previous work has found little difference in results when limiting the outcome to a primary diagnosis of COVID-19 (rather than including any hospitalisation/death after a positive test), it is possible that changes in this bias may have occurred over time and could mean comparisons of inequalities in COVID-19 over waves may be subject to bias."

Discussion:

R2.2. miss in the discussion is to describe the results in the context of previous studies. You mention in the introduction why ethnical inequalities in COVID may be present; and why socioeconomic inequalities may be present, but in the discussion the reader may want to know whether these results agree or disagree with previous studies and why that may be.
A: We thank you for this point and have added the following: *"We add to previous research suggesting that socio-economic disadvantage and occupation operates alongside ethnicity in shaping the risk adverse COVID-19 outcomes, and draw specific attention to the possibility that vulnerability to these factors (rather than, or in addition to, exposure) may differ by ethnicity.[13,22]"*

That's all. Thanks for the paper.
We are pleased for your comments and suggestions.
